# Near-real-time monitoring of global CO_2_ emissions reveals the effects of the COVID-19 pandemic

**DOI:** 10.1038/s41467-020-18922-7

**Published:** 2020-10-14

**Authors:** Zhu Liu, Philippe Ciais, Zhu Deng, Ruixue Lei, Steven J. Davis, Sha Feng, Bo Zheng, Duo Cui, Xinyu Dou, Biqing Zhu, Rui Guo, Piyu Ke, Taochun Sun, Chenxi Lu, Pan He, Yuan Wang, Xu Yue, Yilong Wang, Yadong Lei, Hao Zhou, Zhaonan Cai, Yuhui Wu, Runtao Guo, Tingxuan Han, Jinjun Xue, Olivier Boucher, Eulalie Boucher, Frédéric Chevallier, Katsumasa Tanaka, Yimin Wei, Haiwang Zhong, Chongqing Kang, Ning Zhang, Bin Chen, Fengming Xi, Miaomiao Liu, François-Marie Bréon, Yonglong Lu, Qiang Zhang, Dabo Guan, Peng Gong, Daniel M. Kammen, Kebin He, Hans Joachim Schellnhuber

**Affiliations:** 1 Department of Earth System Science, Tsinghua University, Beijing, China; 2 Laboratoire des Sciences du Climat et de l’Environnement LSCE, CEA CNRS UVSQ, Centre d’Etudes Orme de Merisiers, Gif-sur-Yvette, France; 3 Department of Meteorology and Atmospheric Science, The Pennsylvania State University, University Park, PA USA; 4 Department of Earth System Science, University of California, Irvine, 3232 Croul Hall, Irvine, CA USA; 5 Division of Geological and Planetary Sciences, California Institute of Technology, Pasadena, CA USA; 6 Jet Propulsion Laboratory, California Institute of Technology, Pasadena, CA USA; 7 Jiangsu Key Laboratory of Atmospheric Environment Monitoring and Pollution Control, Collaborative Innovation Center of Atmospheric Environment and Equipment Technology, School of Environmental Science and Engineering, Nanjing University of Information Science & Technology, Nanjing, China; 8 Key Laboratory of Land Surface Pattern and Simulation, Institute of Geographical Sciences and Natural Resources Research, Chinese Academy of Sciences, Beijing, China; 9 Climate Change Research Center, Institute of Atmospheric Physics, Chinese Academy of Sciences, Beijing, China; 10 Key Laboratory of Middle Atmosphere and Global Environment Observation, Institute of Atmospheric Physics, Chinese Academy of Sciences, Beijing, China; 11 School of Environment, Tsinghua University, Beijing, China; 12 School of Mathematical School, Tsinghua University, Beijing, China; 13 Department of Mathematical Sciences, Tsinghua University, Beijing, China; 14 Center of Hubei Cooperative Innovation for Emissions Trading System, Wuhan, China; 15 Faculty of Management and Economics, Kunming University of Science and Technology, 13 Kunming, China; 16 Economic Research Centre of Nagoya University, Furo-cho, Chikusa-ku, Nagoya, Japan; 17 Institut Pierre-Simon Laplace, Sorbonne Université / CNRS, Paris, France; 18 Université Paris Dauphine, Place du Maréchal de Lattre de Tassigny, 75016 Paris, France; 19 Center for Global Environmental Research, National Institute for Environmental Studies, Tsukuba, Japan; 20 Center for Energy and Environmental Policy Research, Beijing Institute of Technology, Beijing, China; 21 Department of Electrical Engineering, the State Key Lab of Control and Simulation of Power Systems and Generation Equipment, Institute for National Governance and Global Governance, Tsinghua University, Beijing, China; 22 Institute of Blue and Green Development Shandong University, Weihai, China; 23 School of Environment, Beijing Normal University, Beijing, China; 24 Institute of Applied Ecology, Chinese Academy of Sciences, Shenyang, China; 25 State Key Laboratory of Pollution Control and Resource Reuse, School of the Environment, Nanjing University, Nanjing, China; 26 Key Laboratory of Wetland Ecology of Ministry of Education, College of Ecology and the Environment, Xiamen University, Xiamen, China; 27 Energy and Resources Group and Goldman School of Public Policy, University of California, Berkeley, CA USA; 28 Potsdam Institute for Climate Impact Research, Potsdam, Germany

**Keywords:** Climate sciences, Atmospheric science, Environmental sciences, Environmental social sciences

## Abstract

The COVID-19 pandemic is impacting human activities, and in turn energy use and carbon dioxide (CO_2_) emissions. Here we present daily estimates of country-level CO_2_ emissions for different sectors based on near-real-time activity data. The key result is an abrupt 8.8% decrease in global CO_2_ emissions (−1551 Mt CO_2_) in the first half of 2020 compared to the same period in 2019. The magnitude of this decrease is larger than during previous economic downturns or World War II. The timing of emissions decreases corresponds to lockdown measures in each country. By July 1st, the pandemic’s effects on global emissions diminished as lockdown restrictions relaxed and some economic activities restarted, especially in China and several European countries, but substantial differences persist between countries, with continuing emission declines in the U.S. where coronavirus cases are still increasing substantially.

## Abstract (web summary)

The COVID-19 pandemic has stopped many human activities, which has had significant impact on emissions of greenhouse gases. Here, the authors present daily estimates of country-level CO_2_ emissions for different economic sectors and show that there has been a 8.8% decrease in global CO2 emissions in the first half of 2020.

## Introduction

Changes in human activities related to the COVID-19 pandemic^1^ affected global energy consumption and associated CO_2_ emissions, but many details of these effects remain unclear. In addition to the considerable differences^2–4^, annual national inventories of energy and fuel use that have historically been used to assess CO_2_ emissions lag reality by 1 or 2 years^5–10^, and are thus not yet available to assess the COVID-19 impacts. This has spurred various efforts for producing more current estimates of emissions changes^11,12^. Initial reports based on a limited sample of power plants and indirect satellite observations of atmospheric pollutants^13,14^ suggested an significant drop in global emissions. The International Energy Agency (IEA) used monthly projections of fossil fuel energy demand to estimate a −5% decline in global CO_2_ emissions in January–April 2020 compared to the same period in 2019^11^. Le Quéré et al. used confinement index data under the assumption that emissions reductions scaled according to mandated lockdown intensity and estimated that daily emissions in April 2020 were 17% less than mean daily emissions in 2019^12^. Yet daily data with detail for all sectors that capture the precise timing of changes in emissions in different regions are lacking (see further discussion in Supplementary Note 1).

Here, we present the estimates of daily, sector-specific, country-level CO_2_ emissions from January 1st, 2019 to June 30th, 2020, constructed primarily from near-real-time activity data, results of the international research initiative Carbon Monitor (https://carbonmonitor.org/). These estimates provide a picture of the daily, weekly, and seasonal dynamics of CO_2_ emissions before and after the COVID-19 pandemic and the economic downturn that it has triggered. For example, the emissions effects of major holidays such as Christmas in the U.S. and Europe, the Spring Festival in China, and Holi Festival in India are evident. Overall, we find an 8.8% (1551 Mt CO_2_) decrease in global CO_2_ emissions in the first half of 2020 related to the COVID-19 pandemic.

## Results

Details of data sources and analytical methods are provided in the “Methods” section. In summary, region- and sector-specific estimates of daily CO_2_ emissions were calculated from hourly datasets of electricity power production in 31 countries, daily vehicle traffic in 416 cities worldwide, daily global passenger aircraft flights and distance flown, monthly production data for industry output in 62 countries, and fuel consumption data combined with weather information for residential and commercial building emissions in 206 countries (see “Methods” for data sources). Our estimates cover fossil and industry sources of global CO_2_ emissions, including process emissions from cement production which were not considered in the IEA assessment^10,11^.

### Near-real-time daily emissions from January 1st, 2019 to June 30th, 2020

**Fig. 1:**
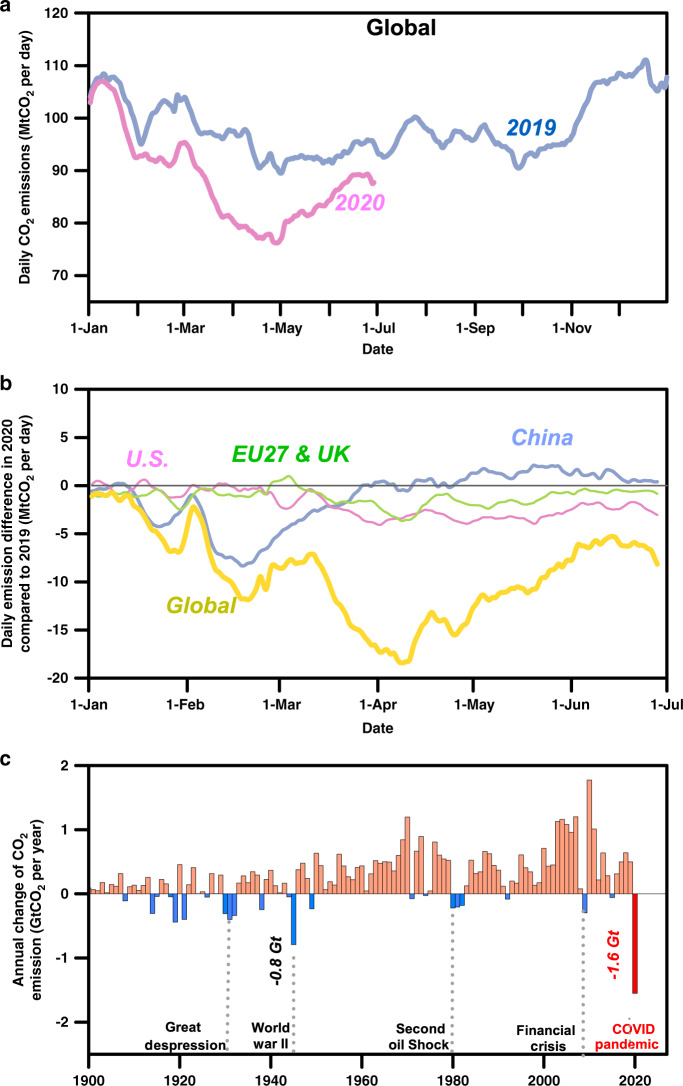
Effects of COVID-19 on global CO_2_ emissions. **a** Daily CO_2_ emissions in 2019 and 2020 (7-day running mean); **b** Global emissions aggregate different timing of effects in different regions (7-day running mean); **c** COVID-19 causes the largest annual decrease of CO_2_ emission since 1900.

Figure 1 shows the substantial COVID-related decreases in CO_2_ emissions between January 1st and June 30th of 2020 as compared to 2019. In the aggregate, emissions were 8.8% lower (1551 Mt CO_2_). The range of seasonal, weekly, and daily variations in CO_2_ emissions in 2019 and January through June of 2020 are remarkably large, as seen in Fig. 1a, b, mainly related to heating and cooling demands inferred from heating and cooling degree days (HDD^15^ and CDD) as well as to periodical seasonal and weekend differences in activities and lower emissions during holidays. For example, the lowest estimated daily CO_2_ emissions in the U.S. was on Christmas Day of 2019, and the lowest daily power-sector CO_2_ emissions in China was during the Spring Festival of 2019. The Spring Festival vacations resulted in a short-term emission drop of similar magnitude than the abnormal impacts of the COVID during the climax of the country’s COVID lockdown in March of 2020. Our monthly averaged estimates of CO_2_ emissions obtained from daily data are consistent with previous studies^16^ and further clarifies the magnitude of the decline of global monthly CO_2_ emissions in February 2019 from decreased energy demand during China’s Spring Festival.

To distinguish the COVID-19 impacts from recurrent seasonal variations and holiday impacts, we estimate the difference between daily emissions in 2020 and the same period in 2019 (Fig. 1 and Supplementary Table S1). Globally, we find an 8.8% (1551 Mt CO_2_) decrease in CO_2_ emissions during the first half of 2020 as compared to the same period in 2019 (including the extra leap day of emissions on February 29th of 2020; light blue vs. pink curves in Fig. 1a). This total difference (1551 Mt CO_2_) is the largest ever decline in emissions over the first half year (Fig. 1c), larger than for any recent economic downturn, and larger than the annual decrease (790 Mt CO_2_) during World War II, although mean emissions were much larger than now at that time. Mean daily emissions over the same period (January–June) were 88.4 Mt CO_2_ per day, which is 10% lower than the daily average emissions in 2019 (98.2 Mt CO_2_ per day). The decline of daily global emissions was the most pronounced in the month of April (−16.9% compared with 2019), but emissions began to recover in late April and May, as economic activities fully resumed in China and parts of Europe (Fig. 1b). In June, power sector emissions were only 1.1% lower in 2020 than 2019, compared to being 9.7% lower in April. However, decreases in mobility-related emissions seem to be more persistent: emissions from ground transportation (data updated to July 31st 2020) were 13.0% lower in July of 2020 than in 2019, though monthly decreases in April and May were much larger (−38.6% and −32.6% respectively) but smaller in June (−15.2%).

It is important to note that the first months of 2020 were exceptionally warm across much of the northern hemisphere, meaning that CO_2_ emissions during that period would have been somewhat lower than in the same period in 2019 even without the disruption in economic activities and energy production caused by COVID-19 and related lockdowns. We made an attempt to attribute the observed power emission reductions to the effects of COVID-19 alone by removing the difference of daily CO_2_ explained by temperature variation between 2019 and 2020 in the winter months (January–March), and comparing the CO_2_ emission in 2020 with the same period in 2019 (See “Methods”). The results of this preliminary attribution analysis suggest that in the winter months (January–March) of 2020, the COVID-19 explains 85% of the power sector reduction, the rest being attributed to warm weather.

**Fig. 2:**
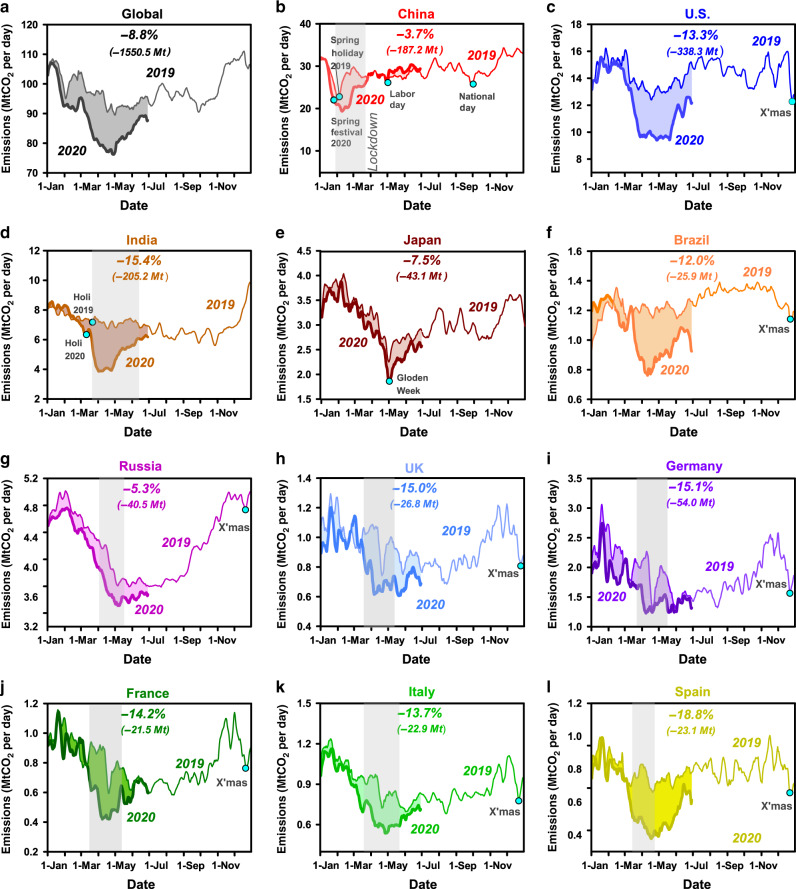
Daily CO_2_ emissions for countries. Effects of the COVID-19 pandemic on daily CO_2_ emissions globally and in each of 11 regions are reflected by the shaded differences between January 1st and June 30th of 2019 and 2020.

Daily CO_2_ emissions time series reveal that the different timings of the reductions were synchronous with lockdown measures. Figure 2 shows trends in daily CO_2_ emissions globally and for 11 major regions. In the first half year of 2020, the most pronounced decline occurred in U.S. (−338.3 Mt CO_2_, −13.3%), followed by EU27 & UK (−205.7 Mt CO_2_, −12.7%), India (−205.2 Mt CO_2_, −15.4%) and China (−187.2 Mt CO_2_, −3.7%), with substantial but progressively decreases in Japan (−43.1 Mt CO_2_, −7.5%), Russia (−40.5 Mt CO_2_, −5.3%) and Brazil (−25.9 Mt CO_2_, −12.0%). The sudden, large, and early drop of Chinese emissions corresponds to the initial outbreak of COVID-19 and to the country’s strict lockdown measures which were gradually relaxed in March. China’s CO_2_ emissions then recovered quickly, however. Monthly relative differences between 2020–2019 were −18.4% in February, −9.2% in March, +0.6% (i.e., greater in 2020 than 2019) in April, and reached +5.4% in May, that is a rebound above 2019 in the same month of the year. The quick recovery of China’s emission has been reported by other studies and is consistent with calculation based on satellite observation^17^. However, the monthly total CO_2_ emission is higher than that of previous year since May 2020 does not necessarily mean the economy has fully recovered. The rebound of economy is normal especially in energy intensive industries, in which the industrial activities and infrastructure construction has suspended during the lockdown, and this could result in the shortage of industrial products and rebound of production after the lockdown is released. In other countries, there was no decrease in emissions due to COVID-19 until late February or March, and the observed drop was coincident with the spread of the virus and onset of lockdowns, with greater decreases in March (U.S.: −14.1%, EU27 & UK: −8.3%, India: −16.9%, Russia: −4.9%, Brazil: −10.8%, Japan: −4.7%) than in February (U.S.: + 1.7%, EU27 & UK: −6.2%, India: +6.4%, Russia: −1.0%, Brazil: −1.4%, Japan −2.2%). In these countries, emissions decreases were the largest in April (U.S.: −25.4%, EU27 and UK: −26.3%, India: −44.2%, Russia: −10.9%, Brazil: −31.3%, Japan: −10.3%). Since May 2020, lockdown restrictions in many of these countries began to ease and emissions deficits became smaller but remained significant (U.S.: −26.4% in May and −14.8% in June; EU27 & UK: −21.6% in May and −6.9% in June; India: −27.6% in May and −15.0% in June; Russia: −8.4% in May and −5.1% in June; Brazil: −26.0% in May and −12.6% in June; Japan: −17.2% in May and −7.6% in June).

**Fig. 3:**
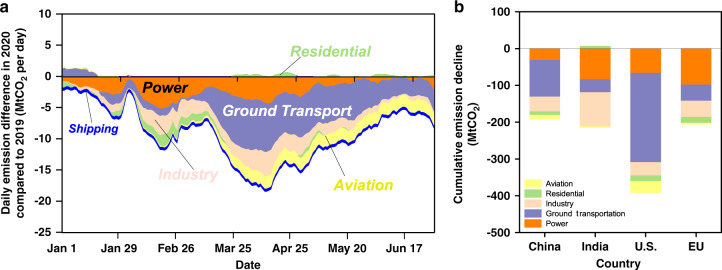
Sectoral effects of COVID-19 on CO_2_ emissions. **a** Sector-specific effects of the COVID-19 pandemic on CO_2_ emissions globally, shown as the 7-day running mean of daily differences between January 1st and June 30th of 2019 and 2020, and **b** the cumulative decline by sectors in each of China, India, U.S., and EU27 & UK in the first half year of 2020.

**Fig. 4:**
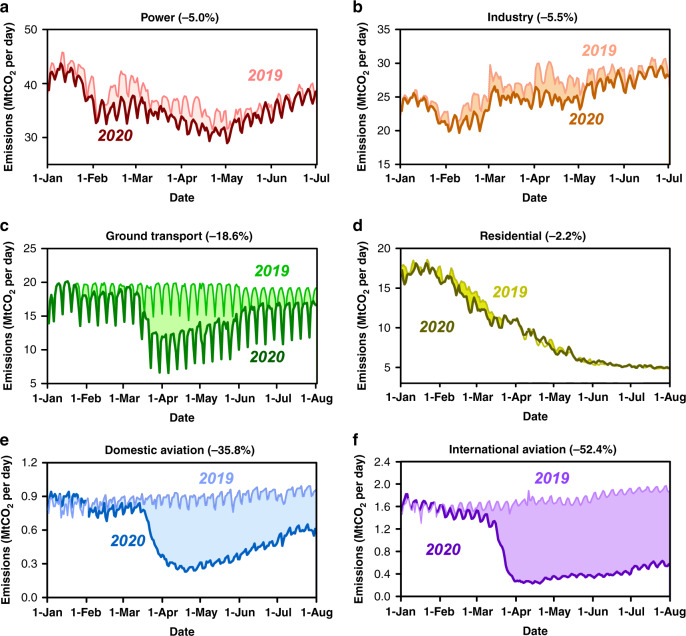
Global daily CO_2_ emissions for sectors. Daily CO_2_ emissions by sectors in 2019 and January 1st through July 1st 2020 for **a** power sector and **b** industry sector, and January 1st through August 1st 2020 for **c** ground transportation sector, **d** residential sector, and aviation sector (**e** domestic aviation; **f** international aviation).

The decrease is mainly due to the ground transportation sector (−18.6%) and domestic (−35.8%) and international aviation (−52.4%) (Figs. 3, 4). Figure 3 shows the breakdown of daily emissions changes by sectors. The largest contributions to the global decrease in emissions in 2020 come from ground transportation (−613.3 Mt CO_2_, 40% of the total decrease; purple in Fig. 3a) and the power sector (−341.4 Mt CO_2_, 22% of the total decrease; orange in Fig. 3a), with somewhat smaller decreases from the industry sector (−263.5 Mt CO_2_, 17% of the total decrease; warm orange in Fig. 3a) and the aviation sector (including domestic aviation and international aviation, −200.8 Mt CO_2_, 13% of the total decrease; yellow in Fig. 3a), and relatively small decreases in international shipping (−89.1 Mt CO_2_, 6% of the total decrease; blue in Fig. 3a) in residential sector emissions, which include residential, public and commercial buildings (−42.5 Mt CO_2_, 3% of the total decrease; green in Fig. 3a). Further details of these sectoral changes (Fig. 4) are discussed below.

### Power Generation

Estimates of power sector emissions rely on near-real-time hourly or daily electricity data. Figure 4a shows that in the first half year of 2020, global CO_2_ emissions from the power sector declined by −5.0% (−341.4 Mt CO_2_), with a small decline in China (−1.4%, −31.3 Mt CO_2_) and somewhat larger decreases in the U.S. (−7.6%, −66.3 Mt CO_2_), India (−12.7%, −83.6 Mt CO_2_) and the EU27 & UK (−19.3%, −98.5 Mt CO_2_) (see also Supplementary Table S2). Some of the drop in China’s power sector emissions was due to warmer winter temperatures in 2020. The negligible differences in emissions during late January and early February of 2020 and 2019 are explained by the different dates of China’s Spring Festival in the two years: 2019 emissions were low during that period due to the festival (Supplementary Fig. S1). The decline of power generation in Qing Ming Festival (April 5th) and the Labor Day holiday (May 1st–5th) are also visible in the results (Supplementary Fig. S1).

### Industry and cement production emissions

Industry emissions from steel, chemicals and other manufactured products from fossil fuel combustion and the cement production process represent on average 29% of the global CO_2_ emissions during a normal year, with a much larger share of national emissions in developing countries, e.g., 39 and 33% in China and India. In this study, only emissions from direct fuel consumption and chemical process emissions by the industry sector were considered whereas electricity-related emissions for industry are counted with the power generation sector. In the first half year of 2020, industry emissions fell by −5.5% globally, incorporating substantial decreases in the China (−2.1%, −40 Mt CO_2_), U.S. (−9.1%, −36.5 Mt CO_2_), the EU27 & UK (−14.1%, −43.5 Mt CO_2_) and India (−22.1%, −92.6 Mt CO_2_) (Fig. 4b and Supplementary Table S3). However, Emissions from China’s steel production (42% of the country’s industrial emissions from fuel combustion in recent years) remained essentially the same as in 2019, with slight increases in January and February (1.4% and 5.0%, respectively) and a modest decrease in March (−1.7%) but rebound in May (4.2%). Overall, despite COVID-19 lockdowns around the world, emissions from China’s steel industry were thus 2.2% higher in the first half year of 2020 than in 2019. In contrast, cement industry emissions (22.2% of China’s industrial emissions from fuel combustion in recent years) decreased substantially. We inferred a −4.8% of cement emissions during the first half year of 2020, composed of a drop of −29.5% drop in January and February combined, of −18.3% in March, but a surge by +3.8%, +8.6% and +8.4% in April, May and June above 2019 values (based on official reports from National Bureau of Statistics^18^).

### Ground transportation emissions

Ground transportation (See “Methods” for data and calculation process) represents 18% of global CO_2_ emissions in recent years. Using TomTom congestion level with daily transportation activity data for 416 global cities in 57 countries, we estimate that, in the first half year of 2020 ground transportation emissions decreased by −18.6% (−613.3 Mt CO_2_), and −17.8% (−685.5 Mt CO_2_) in the first 7 months of 2020 (Fig. 4c, Supplementary Fig. S2 and Supplementary Table S4). As lockdowns began in China, monthly ground transport emissions in January 2020 were lower by −18.6% compared to 2019, by −53.8% decrease in February, and emissions deficits in this sector were progressively reduced in March (−25.0%), April (−16.1%), May (−10.8%), June (−5.9%), and July (−4.2%) as restrictions were relaxed. As with other sectors, the largest decreases in ground transport emissions in other countries than China occurred later. Ground transportation emissions in the EU27 & UK and India dropped by −16.7% and −25.7% in March, respectively, with decreases reaching −31.9% and −65.6% in April, respectively, before shrinking somewhat in May (−20.7% and −34.4%, respectively), June (−1.2% and −16.7%, respectively), and July (−0.3% and −7.2% respectively). In EU27 & UK, decreases in over the first seven months of 2020 were largest in Spain (−16.6%), Italy (−13.7%), France (−13.0%), and the UK (−12.4%) (Supplementary Fig. S2). Even without national restrictions to transportation in U.S., Brazil, and Japan, ground transportation in those countries dropped by 24%, −17.5%, and −7.7%, respectively, in the first 7 months of 2020.

### Aviation and shipping emissions

Emissions from global aviation decreased by −43.9% (−200.8 Mt CO_2_) and −46.7% (254.5 Mt CO_2_) during the first half year and the first 7 months of 2020 respectively, of which roughly 70% of the drop was related to international flights (Fig. 4e, f and Supplementary Table S5). Decreases in emissions from international flights are included in our global estimates, but only domestic flight emissions were attributed to different countries, based on the departure and arrival country for each flight (See “Methods”). The total number of flights and global aviation emissions show two big decreases, one in Asia near the end of January and another coincident with travel bans and lockdown measures in the rest of the world that began in the middle of March. Global aviation emissions began rebounding somewhat in late April and have slightly and gradually increased throughout the end of July. However, international flight emissions in July 2020 were still 72.0% lower than the emissions in July 2019. Emissions from international shipping were 25% lower in the first half year of 2020 than over the same period in 2019.

### Commercial and residential buildings

Due to COVID-19 pandemic, more people stayed at home. In this section, we analyze changes of emissions from residential fuel use, while the change in electricity consumption by households and commercial/public buildings was counted as part of the power sector emissions. Obtaining daily emissions from this sector is more uncertain than from other sectors, given that daily residential natural gas consumption data is not available for all countries. We looked at publicly available natural gas daily consumption data by residential and commercial buildings for France (https://www.smart.grtgaz.com/fr/consommation) during 2019 and 2020 as a case study to show the change in residential natural gas consumption (see “Methods”). In this country, residential emissions did not change from other factors than heating degree days variations in 2020, even though most people were confined at home. We extrapolated this behavior to other countries by assuming that emissions from fuel use (oil and gas) in residential and commercial buildings varied only from population-weighted heating degree days calculated from surface (2-m) air temperature data for 206 countries^19^, taking into account country-specific weekly cycles and holidays. Global heating demand in the first seven months of 2020 was down by −2.1% compared to 2019, owing to the abnormally warm northern-hemisphere winter condition^20^, resulting in a proportional decrease in emissions from this sector. We stress gain the fact that this estimate rests on the assumption that residential and commercial fuel consumption was driven mostly by temperature, with no substantial change in the intrinsic fuel consumption of households and commercial buildings during the lockdown period, an assumption which remain to be tested as residential energy use data will be available later this year.

### Observation and verification from air quality data

Our estimates of decreases in fossil and industry CO_2_ emissions are consistent with observed changes in nitrogen dioxide (NO_2_) emissions, which are also mainly produced by fossil fuel combustion. For example, tropospheric NO_2_ column concentration data from satellites^21,22^, and surface NO_2_ concentrations from air quality stations show decreases (See “Methods”, Supplementary Fig. S3 and Supplementary Table S6) consistent with our estimates of reductions in fossil fuel and industry emissions. In China, decreases in NO_2_ in the first four months of 2020 are consistent with our calculated NO_2_ emission declines based on near-real-time activity and emission data (See Methods and Supplementary Table S6). Substantial and similar decreases in NO_2_ occurred over the U.K., France, Germany, Italy, and the U.S. Over India, the observed decline in NO_2_ was weaker, also consistent with our emissions estimates.

Overall, NO_2_ declines over China in January and February are the largest declines since the beginning of the OMI satellite record in 2004. The consistent results from both ground-based and satellite monitoring systems confirm the significant decline of the NO_2_ concentrations due to COVID-19 (See “Methods”). Based on the OMI satellite data, Over the U.K., France, Germany, and Italy, NO_2_ decreased by a similar amount than in the U.S. Over India, NO_2_ showed a weaker decline, also consistent with satellite data.

## Discussion

Our estimates of daily CO_2_ emissions reveal the effects of COVID-19 on human energy use and CO_2_ emissions in the first half year of 2020 with sectoral information of daily emission estimates till the end of July, and how emissions decreases developed in time across countries. The longer-term effects of the pandemic on emissions remain uncertain, and depend upon factors such as the efficacy and stringency of public health policies, the recovery of economies and human activities, and persistent changes in human behavior. Nevertheless, our data indicate a fast recovery in most countries by the end of June except in the U.S., Brazil, and India where the number of COVID cases continued to remain high. In China, we even observed a rebound of emissions above the levels of 2019 as early as the beginning of May.

If the pandemic remains under control in the next few months, the decrease of annual emissions will be considerably less than during the first half of the year. We estimate that total global CO_2_ emissions were 8.8% lower in the first half year of 2020 than in 2019. Based on an assumption that COVID-19 outbreaks will fade everywhere in the second half of the year, the International Monetary Fund predicted that global economic output (GDP) will decrease by −3.0% in 2020^1,23^. In comparison, our results for June show that emissions nearly recovered in different countries after lockdown measures were relaxed. For example, daily CO_2_ emissions rebounded in most countries since April or May, with China’s emissions in May of 2020 exceeding its emissions in May of 2019 by 5.4%. Yet decreases in emissions remain substantial and even amplified in some countries that remained affected by a high number of COVID cases. For example, U.S. emissions were 14.8% lower in June of 2020 than in June of 2019, even though lockdowns were at the same time being relaxed throughout the country.

We emphasized that the absolute decreases in CO_2_ emissions are larger than any in history, including those that occurred during the recent 2008–2009 global financial crisis. At face value, an 8.8% relative reduction of emissions seems to be small when compared to the magnitude and extent of the disturbance of human activities that the COVID produced. This means that the long-term emissions decreases needed in this century to achieve low arming targets must be based on structural and transformational changes in energy production systems, de-carbonization of transportation and improved building energy use efficiency, that is an improvement of the carbon intensity of economies rather than decreases of human activities.

Thus, as we continue to update daily emissions, it will be possible to monitor subtle changes in activity and to detect slower changes in behavior and infrastructure. For example, the longer-term effects of the pandemic on transportation emissions may reflect both a legacy decrease in activity (e.g., if more people continue to work from home) and modal changes (e.g., if urban commuters avoid mass transit), trends that we can now observe over time. Similarly, the method we developed will allow us to assess persistent changes in the carbon intensity of countries’ economies (CO_2_ emissions per unit GDP) as lockdown restrictions are relaxed, activities resume, and post-pandemic policies are adopted. For example, given disproportionate impacts on activities such as cement production, we already showed substantial reductions in the emissions per unit of GDP over in the first half year of 2020 (−2.1% in China, −4.5% in EU, and −9.0% in the U.S.). However, although numerous organizations and some policy-makers have now emphasized the opportunity for a Green Recovery that will both revive economies and advance climate goals^24,25^, emissions could also rebound and exceed pre-pandemic levels if recovery and stimulus rely on carbon-intensive energy availability. And it is here that the value of near-real-time monitoring to policy making becomes apparent: ambitious climate goals, such as limiting the increase in mean global temperatures to 1.5 °C, leave no time for such a Carbon Rebound^26^. It is thus critical that structural changes can be detected as soon as possible, in order to identify and possibly modify policies that are less effective. In the future, our near-real estimates of emissions could shorten the response time of policy adjustments by roughly a year (compared to annual emissions estimates). The detail and timeliness of our emissions estimates will therefore facilitate more agile and adaptive management of CO_2_ emissions during both the pandemic recovery and the ongoing energy transition.

## Methods

### Annual total and sectoral emissions per country in the baseline year 2019

The CO_2_ emissions and sectoral structure in 2018 for countries and regions are extracted from EDGAR V5.0^7^, and the emissions are scaled to the year 2019 based on the growth rates from Liu et al.^27^ and studies by the Global Carbon Project^28,29^. For countries with no current estimates of emission growth rates in 2019 such as Russia, Japan, and Brazil, we assume their growth rates of emissions were 0.5% based on the emission growth rates of the rest of world^28^.

We calculated CO_2_ emissions based on the methodology developed previously^3^:

1 $$Emis = {\sum} {{\sum} {{\sum} {AD_{i,j,k} \cdot EF_{i,j,k}} } }.$$

*i, j, k* reflect the regions, sectors, and fuel types respectively. In our calculation, index *i* covers countries, index *j* covers four sectors that are power generation, industry, transportation and household consumption, while *k* covers three primary fossil fuel types which are coal, oil and natural gas. Emission factors can be further separated into the net heating values for each fuel “v”, the energy obtained per unit of fuel (TJ/t), the carbon content “c” (tC/TJ) and the oxidation rate “o”, which is the fraction (in %) of fuel oxidized during combustion and emitted to the atmosphere.

2 $$Emis = {\sum} {{\sum} {{\sum} {AD_{i,j,k} \cdot \left( {v_{i,j,k} \cdot c_{i,j,k} \cdot o_{i,j,k}} \right)} } }.$$

Given the large uncertainty of CO_2_ emission in China^4,30^, we calculated China’s CO_2_ emissions separately. For China, the energy consumption of coal, oil and gas in 2000–2017 are based on energy balance tables from China Energy Statistical Yearbook^31^. However, due to the 2 years lag of the publications of China Energy Statistical Yearbook, we project the energy consumption of coal, oil and gas in 2018 and 2019 by multiplying the annual growth rates of coal, oil, and gas reported on the Statistical Communiqué^32^. Country-specific emission factors are adopted in the calculation, which are relatively lower to IPCC default emission factors^3,33^.

We assumed that the emission factors and the structure remain unchanged for each country in 2020 when comparing with 2019. Thus, the rate of change of the emission is calculated based solely on the change of the energy consumption data in 2020 compared to the same period of 2019.

Based on the assumption of sectoral carbon intensity and energy structure remaining unchanged from 2018, the EDGAR sectors were aggregated into several main sectors, including power sector, ground transport sector, industry sector, residential sector, aviation sector, and international shipping sector.

### Power sector

For China, we used daily thermal generation data in China till May 24, 2020, and extend to the end of June by linear interpolation with daily coal consumption of six power enterprises from the WIND (https://www.wind.com.cn/). For India, daily total electricity generation data by production types are acquired from Power System Operation Corporation Limited (https://posoco.in/reports/daily-reports/), and we calculated the thermal production by aggregating the electricity produced by *Coal*, *Lignite*, and *Gas, Naphtha and Diesel*. For the U.S., we calculated daily thermal production by summarizing the electricity produced by coal, petroleum and gas of 48 states from the Energy Information Administration’s Hourly Electric Grid Monitor (https://www.eia.gov/beta/electricity/gridmonitor/). For EU countries and UK, electricity generation data by production types at resolution of 1 h to 15 min are collected from ENTSO-E Transparent platform (https://transparency.entsoe.eu/dashboard/show). Data of the 24 EU countries (Austria, Belgium, Bulgaria, Cyprus, Czech Republic, Denmark, Estonia, Finland, France, Germany, Greece, Hungary, Italy, Ireland, Latvia, Lithuania, Netherlands, Poland, Portugal, Romania, Slovakia, Slovenia, Spain, Sweden) and United Kingdom are available from the data platform. We remove the outliers and fill the N/A values by using “interpolate()” function built in Python Pandas packages, then aggregate the thermal production data into daily level. For Russia, hourly electricity generation data are collected from the United Power System of Russia (http://www.so-ups.ru/index.php). For Japan, we collect the hourly electricity generation data by production types from OCCTO (Organization for Cross-regional Coordination of Transmission Operators, https://www.occto.or.jp/) whose data are collected from ten electricity providers in Japan (Hokkaido Electric Power Network, Tohoku Electric Power Network, Tokyo Electric Power Company, Chubu Electric Power Grid, Hokuriku Electric Power Company, Kansai Electric Power, Chugoku Electric Power Transmission & Distribution, Shikoku Electric Power Company, Kyushu Electric Power Transmission & Distribution, and Okinawa Electric Power Company). For Brazil, hourly electricity generation data by production types are acquired from the Operator of the National Electricity System (http://www.ons.org.br/Paginas/). We calculate the national emission changes based on the changes of daily thermal production, or total electricity generation data when thermal production data are not available (i.e., Russia).

For countries not listed above, we estimate the emission changes in 2020 based on the start time of the national closures. Firstly, as the aforementioned countries account for 74% of the global total emissions in the power sector, we disaggregate the global power emissions in 2019 by the weight of the daily totals of emissions of aforementioned countries into daily level, and then calculate the daily emissions of the rest of the world by subtracting the daily totals of aforementioned countries from daily global power emissions. Secondly, we collect the periods of national closure from Wikipedia^34^ and related news. Based on the daily emission estimates of the power sector in this study, we use the average emission change rate during the lockdown period of the aforementioned countries with national closures, to estimate the emission changes of countries in the rest of the world during their national closures. Then we aggregate the daily emission changes of the rest of the world and calculate the daily total reduction rate caused by closures compared with the same day in previous year.

We have tried to correct the power generation data for the temperature effect, i.e., power generation change caused by temperature change from year 2019 to year 2020. The temperature correction is conducted in two steps. Step 1: finding the relationship between daily power generated and daily temperature. This was achieved by establishing a linear regression between daily power generation data and daily temperature data for each country. When a medium to strong correlation between power generation and temperature is found (regression coefficient *R*^2^ > 0.5), step 2 (temperature correction) is conducted subsequently. Step 2: We use the linear regression coefficients and the temperature change between year 2020 and year 2019 (temperature difference on the same day of these 2 years) to calculate the corrected power generation for year 2020.

### Industry and cement production

For China, the industrial sector was divided into four sub-categories including steel industry, cement industry, chemical industry, and other industries, based on the structure of industrial emissions calculations conducted by IEA^10^. For each category, the monthly production data was obtained and the corresponding month-on-month growth rate was calculated accordingly. Specifically, the production data was regarded as the emission estimator i.e., activity while the emission factors were assumed the same as 2019 for each industry. For the steel industry, we collected the global monthly crude steel production data from the World Steel Association website (https://www.worldsteel.org/) while the monthly production data of cement, chemicals as well as other industries were referred to the National Bureau of Statistics website. For the latter two multi-component categories, the production of sulfuric acid, caustic soda, soda ash, ethylene, chemical fertilizer, chemical pesticide, primary plastic and synthetic rubber was taken into account while 26 other industrial products including crude iron ore, phosphate ore, salt, feed, refined edible vegetable oil, fresh and frozen meat, milk products, liquor, soft drinks, wine, beer, tobaccos, yarn, cloth, silk and woven fabric, machine-made paper and paperboards, plain glass, ten kinds of nonferrous metals, refined copper, lead, zinc, electrolyzed aluminum, industrial boilers, metal smelting equipment, and cement equipment were included in the other industries sub-group. The calculation of growth rate for the steel and cement industries was relatively straightforward, in that the 2020 and 2019 month-on-month data were compared. In terms of the latter two multi-component groups, the growth rates were evaluated based on the weighted contribution from each product. Based on the emission distribution of these four industries in the industrial sector in China in year 2019 and the growth rates obtained as stated above, we finally estimated the monthly emission in the first half year of 2020.

For US, EU27 & UK, India, Russia, Japan, and Brazil, we use the cumulative Industrial Production Index to estimate the growth rates of emissions in these countries or regions, collected from the U.S. Federal Reserve Board (https://www.federalreserve.gov), Eurostat (https://ec.europa.eu/eurostat/home), Japan Ministry of Economy, Trade and Industry (https://www.meti.go.jp), Russia Federal State Statistics Service (https://eng.gks.ru), India Ministry of Statistics and Programme Implementation (http://www.mospi.nic.in) and Brazilian Institute of Geography and Statistics (https://www.ibge.gov.br/en/institutional/the-ibge.htm) respectively. However, the last observations in EU27 & UK and India were in May 2020. To estimate the current growth in June 2020, for EU27 & UK and India, we adopt the predicted results from Trading Economics (https://tradingeconomics.com). Based on the growth rates, we calculate the monthly data of the industrial sector for 2019 and the first half year of 2020. The monthly industrial emissions are allocated to daily emissions by daily thermal production data. We follow the same measure for the power sector to calculate the emission from industry and cement production for the rest of the world.

### Ground transportation

We collected TomTom congestion global level data from TomTom website (https://www.tomtom.com/en_gb/traffic-index/). The congestion level (called *X* hereafter) represents the extra time spent on a trip, in percentage, compared to uncongested conditions. TomTom congestion level data are available for 416 cities across 6 continents and 57 countries at a temporal resolution of one hour to 15 min. More than half of the cities reported by TomTom are in Europe  (228 cities from 30 countries, Russia not included) and North America (93 cities), while 59 cities are from 13 countries in Asia (including Russia), 13 cities are from 5 countries in South America, and 23 cities are from Africa and Oceania. The list of 416 cities includes most of the major cities in these countries. Of note that a zero congestion level means that the traffic is fluid, but rather than no cars and zero emissions. It is thus important to identify the low threshold of emissions when the congestion level is zero. We compared the time series of daily mean TomTom congestion level with the daily mean car counts (called *Q* hereafter) on main roads in Paris. The daily mean car counts were reported by the City’s service (https://opendata.paris.fr/pages/home/). We used a sigmoid function to describe the relationship between *X* and *Q* (Supplementary Fig. S4):

3 $$Q = a + \frac{{bX^c}}{{d^c + X^c}}.$$

where *a*, *b*, *c*, and *d* are the regression parameters. It is shown that the regression can reflect large drop down in the ground transportation due to the lockdown and the recovery afterwards. We assume that the daily emissions were proportional to this relative magnitude of daily mean car counts. Then, we applied the regression built for Paris to other cities included in the TomTom dataset, assuming that the relative magnitude in car counts (and thus emissions) follow the similar relationship with TomTom. We compared the time series of TomTom congestion level in the first quarter of 2019 and 2020. The emission changes were first calculated for individual cities, and then weighted by city emissions to aggregate to national changes. The weighting emissions are taken from the gridded EDGARv4.3.2 emission map for the “road transportation” sector (1A3b) (https://edgar.jrc.ec.europa.eu/) for the year 2010, assuming that the spatial distribution of ground transport does not change significantly within a country. However, during the COVID-19 pandemic, the pattern of inner-city and inter-city ground transportation may change differently due to the lockdown measures. But this asymmetric change of ground transportation may not be effectively captured by the TomTom congestion level data, since they mainly represent the transportation conditions within cities. For countries not included in the TomTom dataset, we assume that the emission changes follow the mean changes of other countries. For example, Cyprus, as an EU member country, is not reported in the TomTom dataset, and its relative emission change was assumed to follow the same pattern of the total emissions from other EU countries included in the TomTom dataset (which covers 98% of EU total emissions). Similarly, the relative emission changes of countries in ROW but were not reported by TomTom were assumed to follow the same pattern of the total emissions from all TomTom countries (which cover 85% of global total emissions). The uncertainty in the TomTom-based *Q*, and thus emissions were quantified by the prediction interval of the regression.

### Aviation

CO_2_ emissions from commercial aviation are usually reconstructed from bottom up emission inventories based on the knowledge of the parameters of individual flights. We calculated the CO_2_ emissions from commercial aviation following this approach. Individual commercial flights are tracked by Flightradar24 (https://www.flightradar24.com) based on reception of ADS-B signals emitted by aircraft and received by their network of ADS-B receptors. As we do not have yet the capability to convert the FlightRadar24 database into CO_2_ emissions on a flight-by-flight basis, we compute CO_2_ emissions by assuming a constant (CO_2_ emission factor per km flown) across the whole fleet of aircraft (regional, narrowbody passenger, widebody passenger, and freight operations). This assumption is reasonable if the flight mix between these categories has not changed significantly between 2019 and 2020.

The International Council on Clean Transportation (ICCT) published that CO_2_ emissions from commercial freight and passenger aviation resulted in 918 Mt CO_2_ in 2018^35^ based on the OAG flight database and emission factors from the PIANO database. IATA estimated a 3.4% increase between 2018 and 2019 in terms of available seat kilometers^36^. In the absence of further information, we consider this increase to be representative of freight aviation as well and use a slightly smaller growth rate of 3% for CO_2_ emissions between 2018 and 2019 to account for a small increase in fuel efficiency. The kilometers flown are computed assuming great circle distance between the take-off, cruising, descent and landing points for each flight and are cumulated over all flights. The FlightRadar24 database has incomplete data for some flights and may miss altogether a small fraction of actual flights, so we scale the ICCT estimate of CO_2_ emissions (inflated by 3% for the year 2019) with the total estimated number of kilometers flown for 2019 (67.91 million km and apply this scaling factor to 2020 data. Again, this assumes that the fraction of missed flights is the same in 2019 and 2020, which seems reasonable. As the departure and landing airports are known for each flight, we can classify the km flown (and hence the CO_2_ emissions) per country, and for each country between domestic or international traffic. The daily CO_2_ emission was computed as the product of distance flown, by a CO_2_ emission factor per km flown.

### International shipping

We obtained daily emissions in 2019 based on the assumption that monthly variation is flat in shipping CO_2_ emissions. In addition, we assume that the change in shipping emissions is linearly related to the change in ship’s volume. We estimated the change of shipment by the first half year by -25% compared to the same period of last year according to the report^37^.

### Residential and commercial buildings

The calculation of emissions was performed in three steps: (1) Calculation of population-weighted heating degree days for each country and for each day based on the ERA5 reanalysis of 2-m air temperature, (2) Using the EDGAR estimates of 2018 residential emissions as the baseline. For each country, the residential emissions were split into two parts, i.e., cooking emissions and heating emissions, according to the EDGAR guidelines. The emissions from cooking were assumed to remain stable, while the emissions from heating were assumed to depend on and vary by the heating demand. (3) Based on the change of population-weighted heating degree days in each country, we scaled the EDGAR 2018 residential emissions to 2019 and 2020. Since the index of heating degree days are daily values, we can get daily emission updates for the residential sources globally. Note that the effect of increased time spent in households on residential buildings and decreased time in commercial and public buildings was not accounted for, since we did not have fuel consumption data for urban areas and building types. Our estimates of residential emissions changes are consistent with those obtained from the City of Paris, based on individual electricity use (https://data.enedis.fr/) and population surveys (Y. Françoise pers. comm.).

We test this assumption by analyzing daily natural gas consumption by commercial and residential buildings in four countries (France, Italy, Belgium, and Spain) for which data was publicly available from national operators. The gas data were converted to CO_2_ emissions using emission factors that account for gas heating content. After removing the effect of temperature using a piecewise linear model, there was no evidence of any substantial difference (either positive or negative) between the calculated emissions and the emissions modeled based on temperature in these countries, suggesting that—despite up to 90% of the population being confined at home in these countries—there was little difference in emissions from residential and commercial buildings. Any decreases in fuel use of commercial buildings may have been compensated by increases in fuel use in households.

### Uncertainty estimates

We followed the 2006 IPCC Guidelines for National Greenhouse Gas Inventories to conduct an uncertainty analysis of the data. First, the uncertainties were calculated for each sector (See Supplementary Table S7 for uncertainty ranges of each sector):

- Power sector: the uncertainty is mainly from inter-annual variability of coal emission factors and changes in mix of generation fuel in thermal production. The uncertainty of power emission from fossil fuel is within (±14%) with the consideration of both inter-annual variability of fossil fuel based on the UN statistics and the variability of the mix of generation fuel (the ratio of electricity produced by coal to thermal production).
- Industrial sector: The uncertainty of CO_2_ from industry and cement production comes from monthly production data. CO_2_ from industry and cement production in China accounts for more than 60% of world total industrial CO_2_, and the uncertainty of emissions in China is 20%. Uncertainty from monthly statistics was derived from 10,000 Monte Carlo simulations to estimate a 68% confidence interval (1 sigma) for China. We calculated the 68% prediction interval of the linear regression models between emissions estimated from monthly statistics and official emissions obtained from annual statistics at the end of each year to deduce the one-sigma uncertainty involved when using monthly data to represent the change for the whole year. The squared correlation coefficients are within the range of 0.88 (e.g., coal production) and 0.98 (e.g., energy import and export data), which indicates that only using the monthly data can explain 88 to 98% of the whole year’s variation^3^; the remaining variation is not covered but reflects the uncertainty caused by the frequent revisions of China’s statistical data after they are first published.
- Ground transportation: The emissions from the ground transportation sector are estimated by assuming that the relative magnitude in car counts (and thus emissions) follow a similar relationship with TomTom congestion index in Paris. The uncertainty in emissions were quantified by the prediction interval of the regression. Applying such a regression to all the 416 cities across the world might introduce additional uncertainties when other cities have a different relationship between *Q* and TomTom congestion level, but this uncertainty is not quantified in this study due to the lack of similar car counts data for a wide range of cities across different countries.
- Aviation: The uncertainty in the aviation sector comes from the difference in daily emission data estimated based on the two methods. We calculate the average difference between the daily emission results estimated based on the flight route distance and the number of flights and then divide the average difference by the average daily emissions estimated by the two methods to obtain the uncertainty in CO_2_ from the aviation sector.
- Shipping: We used the uncertainty analysis from IMO as our uncertainty estimate for shipping emissions. According to the Third IMO Greenhouse Gas study 2014^38^, the uncertainty in shipping emissions was 13% based on bottom-up estimates.
- Residential: The 2-sigma uncertainty in daily emissions is estimated as 40%, which is calculated based on a comparison with daily residential emissions derived from real fuel consumption in several European countries, including France, Great Britain, Italy, Belgium, and Spain.

The uncertainty in the emission projection for 2019 is estimated as 2.2% by combining the reported uncertainty of the projected growth rates and the EDGAR estimates in 2018.

Then, we combine all the uncertainties by following the error propagation equation from the IPCC. Equation 4 is used to derive the uncertainty of the sum and could be used to combine the uncertainties of all sectors:

4 $$U_{total} = \frac{{\sqrt {{\sum} {(U_s \cdot \mu _s)} } }}{{\left| {{\sum} {\mu _s} } \right|}},$$

where *U*_*s*_ and *μ*_*s*_ are the percentage and quantity (daily mean emissions) of the uncertainty of sector *s*, *s* respectively. Equation 5 is used to derive the uncertainty of the multiplication, which in turn is used to combine the uncertainties of all sectors and of the projected emissions in 2019:

5 $$U_{overall} = \sqrt {{\sum} {U_i^2} }.$$

### Satellite observation and data sources

To validate the response of the atmosphere, including CO_2_ concentration and air quality, to the decreased fossil fuel burning and transportation, we collected NO_2_, aerosol optical depth (AOD) and column-averaged dry air mole fraction of CO_2_ (XCO_2_) data from satellites (NO_2_ from OMI, AOD from MODIS, and XCO_2_ from GOSAT) and surface daily average nitrogen dioxide (NO_2_, μg/m^3^), carbon monoxide (CO, μg/m^3^) from 1600 air quality monitoring sites (China and US, Supplementary Fig. S3) in to investigate the impact of COVID-19 on air quality and atmospheric CO_2_.

Surface air quality data in China were collected from the daily report by the Ministry of Ecology and Environment of China (http://www.mee.gov.cn/). Measurements of daily average nitrogen dioxide (NO_2_, μg/m^3^), carbon monoxide (CO, μg/m^3^), and particulate matter smaller than 2.5 μm (PM_2.5_, μg/m^3^) from 1580 sites used to estimate pollution changes between the first quarters of 2019 and 2020. Surface air quality data in the U.S. is downloaded from the Air Quality System operated by the U.S. Environmental Protection Agency (https://www.epa.gov/aqs). Measurements of daily maximum 1-h NO_2_ (ppb), daily maximum 8-h CO (ppm), and daily average PM_2.5_ (μg/m^3^) from 983 sites are used. For March 2020, data availability is limited in the U.S. with 20 sites for NO_2_, 31 for CO, and 309 for PM_2.5_. Sites with missing data for NO_2_/CO (PM_2.5_) at over 20 (5) days in any months will be excluded.

We obtained monthly NO_2_ data from the Ozone Monitoring Instrument (OMI) provided by Tropospheric Emission Monitoring Internet Service, which has a spatial resolution of 0.125° × 0.125° and a temporal coverage from October 2004 to March 2020. We only included the data from January 2013 to May 2020 in the work (Supplementary Figs. S5–S8). For AOD, we chose daily Level 2 MOD 04 data from MODIS^39^ and then calculated the monthly averaged AOD from January 2013 to March 2020. Only “good” and “very good” data (in AOD_550_Dark_Target_Deep_Blue_Combined_QA_Flag 2 and 3) were kept in the calculation (Supplementary Fig. S9). At last, we calculated the monthly XCO_2_ data with a resolution of 2.5° × 2.5° from the Greenhouse Gases Observing Satellite “IBUKI” (GOSAT). Because of the delay in the data processing at National Institute for Environmental Studies (NIES), we used a bias-uncorrected version V02.81 for the period of January 2013 to May 2020. With the consideration of the focus on an abnormal event due to COVID-19, the bias-uncorrected data is proper for this study.

All of the monthly averaged data were re-gridded to 1° × 1°. We focused on four emitting regions, China, U.S., EU4 (UK, France, Germany, and Italy), and Indian, and then calculated the country level monthly averaged NO_2_, AOD, and XCO_2_ values.

Surface air pollution in China was significantly reduced during the epidemic period (Supplementary Fig. S3). A deep reduction of NO_2_ by 31.7% was observed on January 24th 2020, one day after the lockdown for many provinces (Supplementary Fig. S3b). The reduction rates were 13.7% for PM_2.5_ and 16.5% for CO on the same day. A clear rebound (U shape) could be found for all pollution after the spring festival (February 5th) in 2019. However, such recovery was missing in 2020 due to the lockdown, leading to a decreasing trend all through the first quarter. On average, pollution concentrations decreased by 23.0% for NO_2_, 15.4% for PM_2.5_, and 12.5% for CO during January–March 2020 relative to the same period in 2019.

Pollution level in the U.S. was also reduced by the epidemic but with smaller magnitude compared to that in China. Surface PM_2.5_ decreased in all first three months in 2020 relative to 2019 with the largest reduction of 20.6% in March. NO_2_ also exhibited large reductions of 9.0% in March 2020 compared to 2019, however, such reduction seemed affected by the limited site numbers (only 20). For example, one site in Salt Lake, Utah reported >200 ppb (normally <40) NO_2_ during March 20–23, 2020. Such episodes were likely caused by fires but weakened the reduction rate of NO_2_ after Middle March (Supplementary Fig. S3e).

Changes of CO were also limited in the U.S., with opposite signs in January and March. Such tendencies could also be biased due to the limited site numbers (only 31).

The observed tropospheric nitrogen dioxide (NO_2_) column concentration data from satellite observation and surface air quality data from ground monitoring networks have exhibited a decrease (Supplementary Table S6) consistent with reduction of fossil carbon fuels emissions.

In China, January, February, March, April, and May 2020 decreased by −32.3%, −34.2%, −4.53%, −3.6%, −12.6%, respectively compared to 2019. Overall, NO_2_ decreased over China by −20.2% from January to May 2020 compared to 2019. In the US, the decrease of NO_2_ first started in February and continued to decrease at least until March 2020. Compared to the same period of the year in 2019, NO_2_ over the U.S. decreased by −23.1% and −14.3% in February and March 2020, respectively (Supplementary Table S6). For the UK, France, Germany, and Italy, we observe similar NO_2_ decreases than over the U.S. India had weaker decline in NO_2_ than other regions.

The decline rate of NO_2_ (−20.2%) based on atmospheric observations can be used to check the consistency of the decrease of NO_2_ emission from the inventory, and given the NO_2_ is mainly contributed by fossil fuel combustion with life time short than one day, the temporal change of NO_2_ emission can could verify the decrease of the fossil fuel combustion and the associated CO_2_ emissions. For China where the most significant decrease of tropospheric NO_2_ column concentration observed, the inventory-based estimates^40^ of power generation (−6.8%), transportation (−37.2%), and industry (−8.1%) are adopted with result of weight mean −23.9% NO_2_ emission in first quarter of 2020 when comparing with 2019. These three sectors together account for 96% of China’s total NO_2_ emissions. The −23.4% decline of the NO_2_ emissions from our bottom-up inventory is consistent with the satellite observed −26% decrease of column NO_2_, and with the −23% decrease of near surface concentrations at the 1680 ground-based stations. For US, the inventory-based estimates of power generation (−4.9%), transportation (−2.7%), and industry(−2.2%) are adopted with result of −2.6% NO_2_ emission in first quarter of 2020 when comparing with 2019, slightly smaller than −4.8% tropospheric NO_2_ column concentration, but difference with the site observation data (−9.0% in March and +0.3% for first quarter), which may be affected by site numbers (only 20 sites in the U.S.). We calculated 1° × 1° monthly mean of NO_2_, AOD, and XCO_2_ from OMI, MODIS, and GOSAT, respectively (Supplementary Table S6).

Here we conservatively considered uncertainty of monthly XCO_2_ as 1.5 ppm. To estimate the uncertainty of changes of 2020 compared to 2019 from January to May, we input above uncertainties of monthly means and run Monte Carlo simulations of 10,000 trials to calculate the 68% confidence intervals (i.e., one sigma range) which are shown in Supplementary Table S6.

## Supplementary information

Supplementary Information

## Data Availability

All data generated or analyzed during this study are included in this article (data available at https://www.carbonmonitor.org or https://www.carbonmonitor.org.cn). AOD from MODIS: https://ladsweb.modaps.eosdis.nasa.gov/missions-and-measurements/products/MOD04_L2/ NO_2_ from OMI: http://www.temis.nl/airpollution/no2col/no2regioomimonth_qa.php XCO_2_ from GOSAT: https://data2.gosat.nies.go.jp/GosatDataArchiveService/usr/download/DownloadPage/view

## Code availability

All code generated during and/or analyzed during the current study are available from the corresponding author upon reasonable request
