# Author Correction: Near-real-time monitoring of global CO_2_ emissions reveals the effects of the COVID-19 pandemic

**DOI:** 10.1038/s41467-020-20254-5

**Published:** 2020-12-02

**Authors:** Zhu Liu, Philippe Ciais, Zhu Deng, Ruixue Lei, Steven J. Davis, Sha Feng, Bo Zheng, Duo Cui, Xinyu Dou, Biqing Zhu, Rui Guo, Piyu Ke, Taochun Sun, Chenxi Lu, Pan He, Yuan Wang, Xu Yue, Yilong Wang, Yadong Lei, Hao Zhou, Zhaonan Cai, Yuhui Wu, Runtao Guo, Tingxuan Han, Jinjun Xue, Olivier Boucher, Eulalie Boucher, Frédéric Chevallier, Katsumasa Tanaka, Yiming Wei, Haiwang Zhong, Chongqing Kang, Ning Zhang, Bin Chen, Fengming Xi, Miaomiao Liu, François-Marie Bréon, Yonglong Lu, Qiang Zhang, Dabo Guan, Peng Gong, Daniel M. Kammen, Kebin He, Hans Joachim Schellnhuber

**Affiliations:** 1 Department of Earth System Science, Tsinghua University, Beijing, China; 2 Laboratoire des Sciences du Climat et de l’Environnement LSCE, CEA CNRS UVSQ, Centre d’Etudes Orme de Merisiers, Gif-sur-Yvette, France; 3 Department of Meteorology and Atmospheric Science, The Pennsylvania State University, University Park, PA USA; 4 Department of Earth System Science, University of California, Irvine, 3232Croul Hall, Irvine, CA USA; 5 Division of Geological and Planetary Sciences, California Institute of Technology, Pasadena, CA USA; 6 Jet Propulsion Laboratory, California Institute of Technology, Pasadena, CA USA; 7 Jiangsu Key Laboratory of Atmospheric Environment Monitoring and Pollution Control, Collaborative Innovation Center of Atmospheric Environment and Equipment Technology, School of Environmental Science and Engineering, Nanjing University of Information Science and Technology, Nanjing, China; 8 Key Laboratory of Land Surface Pattern and Simulation, Institute of Geographical Sciences and Natural Resources Research, Chinese Academy of Sciences, Beijing, China; 9 Climate Change Research Center, Institute of Atmospheric Physics, Chinese Academy of Sciences, Beijing, China; 10 Key Laboratory of Middle Atmosphere and Global Environment Observation, Institute of Atmospheric Physics, Chinese Academy of Sciences, Beijing, China; 11 School of Environment, Tsinghua University, Beijing, China; 12 School of Mathematical School, Tsinghua University, Beijing, China; 13 Department of Mathematical Sciences, Tsinghua University, Beijing, China; 14 Center of Hubei Cooperative Innovation for Emissions Trading System, Wuhan, China; 15 Faculty of Management and Economics, Kunming University of Science and Technology, 13, Kunming, China; 16 Economic Research Centre of Nagoya University, Furo-cho, Chikusa-ku, Nagoya, Japan; 17 Institut Pierre-Simon Laplace, Sorbonne Université/CNRS, Paris, France; 18 Université Paris Dauphine, Place du Maréchal de Lattre de Tassigny, 75016 Paris, France; 19 Center for Global Environmental Research, National Institute for Environmental Studies, Tsukuba, Japan; 20 Center for Energy and Environmental Policy Research, Beijing Institute of Technology, Beijing, China; 21 Department of Electrical Engineering, the State Key Lab of Control and Simulation of Power Systems and Generation Equipment, Institute for National Governance and Global Governance, Tsinghua University, Beijing, China; 22 Institute of Blue and Green Development Shandong University, Weihai, China; 23 School of Environment, Beijing Normal University, Beijing, China; 24 Institute of Applied Ecology, Chinese Academy of Sciences, Shenyang, China; 25 State Key Laboratory of Pollution Control and Resource Reuse, School of the Environment, Nanjing University, Nanjing, China; 26 Key Laboratory of Wetland Ecology of Ministry of Education, College of Ecology and the Environment, Xiamen University, Xiamen, China; 27 Energy and Resources Group and Goldman School of Public Policy, University of California, Berkeley, CA USA; 28 Potsdam Institute for Climate Impact Research, Potsdam, Germany

**Keywords:** Climate sciences, Atmospheric science, Environmental sciences, Environmental social sciences

Correction to: *Nature Communications* 10.1038/s41467-020-18922-7, published online 14 October 2020.

The original version of this Article contained an error in the spelling of the author Yiming Wei, which was incorrectly given as Yimin Wei.

This has now been corrected in both the PDF and HTML versions of the Article.

