# Supplementary material for: Near-real-time monitoring of global CO2 emissions reveals the effects of the COVID-19 pandemic
Source: Nat Commun. 2020 Oct 14;11:5172. doi: 10.1038/s41467-020-18922-7 (PMC7560733; doi:10.1038/s41467-020-18922-7)
Supplement: Supplementary file 1 — Supplementary Information [file 41467_2020_18922_MOESM1_ESM.pdf]

Supplementary Information

Near-real-time monitoring of global CO_2 emissions reveals the effects of the COVID-19 pandemic

Zhu Liu[1*†], Philippe Ciais[2†], Zhu Deng[1†], Ruixue Lei[3], Steven J. Davis[4], Sha Feng[3], Bo Zheng[2], Duo Cui[1], Xinyu Dou[1], Biqing Zhu[1], Rui Guo[1], Piyu Ke[1], Taochun Sun[1], Chenxi Lu[1], Pan He[1], Yuan Wang[5,6], Xu Yue[7], Yilong Wang[8], Yadong Lei[9], Hao Zhou[9], Zhaonan Cai[10], Yuhui Wu[11], Runtao Guo[12], Tingxuan Han[13], Jinjun Xue[14, 15, 16], Olivier Boucher[17], Eulalie Boucher[17], Frédéric Chevallier[2], Katsumasa Tanaka[2,27], Yimin Wei[18], Haiwang Zhong[19], Chongqing Kang[19], Ning Zhang[20], Bin Chen[21], Fengming Xi[22], Miaomiao Liu[23], François-Marie Bréon[2], Yonglong Lu[24], Qiang Zhang[1], Dabo Guan[1], Peng Gong[1], Daniel M. Kammen[25], Kebin He[11] and Hans Joachim Schellnhuber[26,1]

1 Department of Earth System Science, Tsinghua University, Beijing, China.
2 Laboratoire des Sciences du Climate et de l'Environnement LSCE, Orme de Merisiers, Gif-sur-Yvette, France
3 Department of Meteorology and Atmospheric Science, The Pennsylvania State University, University Park, PA, USA
4 Department of Earth System Science, University of California, Irvine, 3232 Croul Hall, Irvine, CA, USA
5 Division of Geological and Planetary Sciences, California Institute of Technology, Pasadena, CA, USA
6 Jet Propulsion Laboratory, California Institute of Technology, Pasadena, CA, USA
7 Jiangsu Key Laboratory of Atmospheric Environment Monitoring and Pollution Control, Collaborative Innovation Center of Atmospheric Environment and Equipment Technology, School of Environmental Science and Engineering, Nanjing University of Information Science & Technology, Nanjing, China
8 Key Laboratory of Land Surface Pattern and Simulation, Institute of Geographical Sciences and Natural Resources Research, Chinese Academy of Sciences, Beijing, China
9 Climate Change Research Center, Institute of Atmospheric Physics, Chinese Academy of Sciences, Beijing, China
10 Key Laboratory of Middle Atmosphere and Global Environment Observation, Institute of Atmospheric Physics, Chinese Academy of Sciences, Beijing, China.
11 School of Environment, Tsinghua University, Beijing, China
12 School of Mathematical School, Tsinghua University, Beijing, China
13 Department of Mathematical Sciences, Tsinghua University, Beijing, China
14 Center of Hubei Cooperative Innovation for Emissions Trading System, Wuhan, China
15 Faculty of Management and Economics, Kunming University of Science and Technology, 13 Kunming, China
16 Economic Research Centre of Nagoya University, Furo-cho, Chikusa-ku, Nagoya, Japan
17 Institut Pierre-Simon Laplace, Sorbonne Université / CNRS, Paris, France
18 Center for Energy and Environmental Policy Research, Beijing Institute of Technology, Beijing, China
19 Department of Electrical Engineering, the State Key Lab of Control and Simulation of Power Systems and Generation Equipment, Institute for National Governance and Global Governance, Tsinghua University, Beijing, China
20 Jinan University, Guangzhou, China
21 Beijing Normal University, Beijing, China
22 Institute of Applied Ecology, Chinese Academy of Sciences, Shenyang, China
23 State Key Laboratory of Pollution Control and Resource Reuse, School of the Environment, Nanjing University, Nanjing, China
24 Key Laboratory of Wetland Ecology of Ministry of Education, College of Ecology and the Environment, Xiamen University, Xiamen, China
25 Energy and Resources Group and Goldman School of Public Policy, University of California, Berkeley, CA, USA
26 Potsdam Institute for Climate Impact Research, Potsdam, Germany
27 Center for Global Environmental Research, National Institute for Environmental Studies, Tsukuba, Japan

* Corresponding authors: zhuliu@tsinghua.edu.cn

† Authors contributed equally

Supplementary Figures

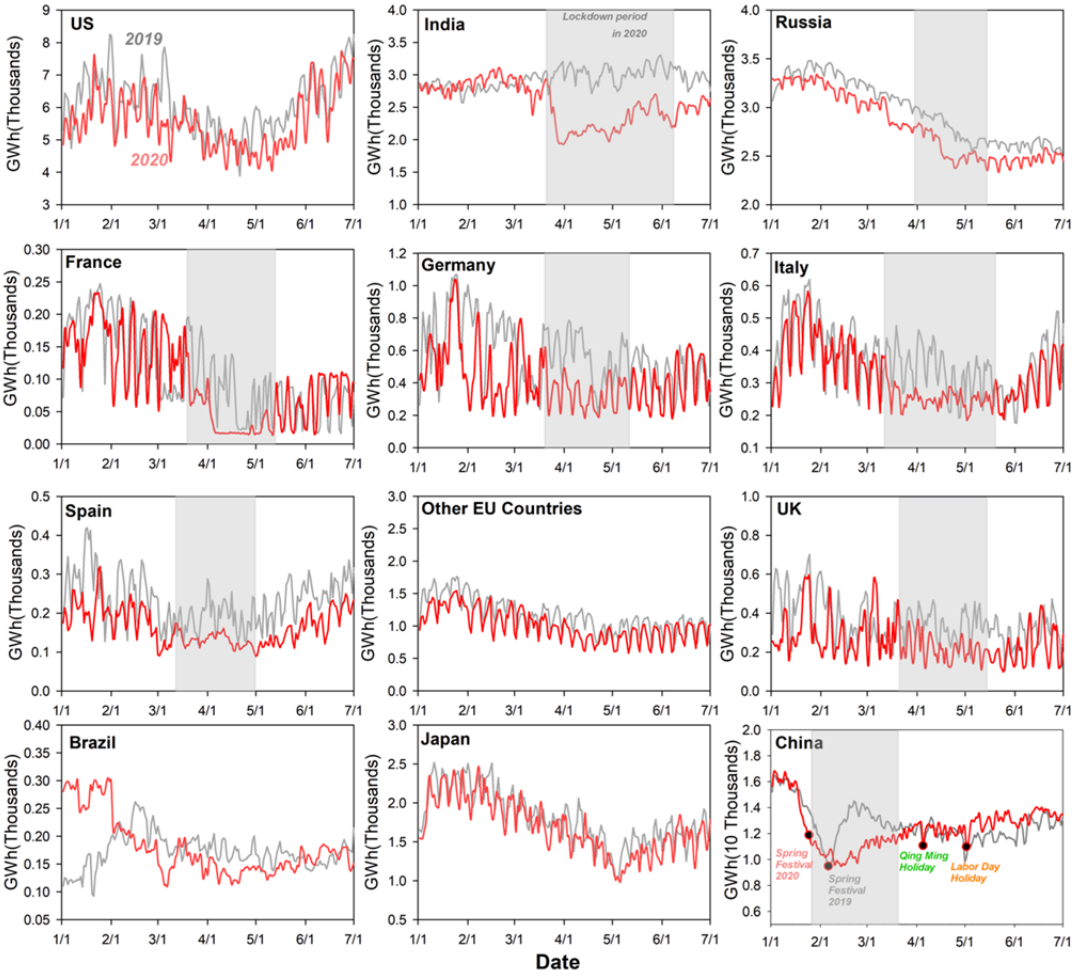

Supplementary Figure 1 | Daily thermal generation for countries. Daily thermal generation (or total electricity generation, i.e. Russia) in 2019 (grey lines) and 2020 (red lines) in the U.S., India, Russia, France, Germany, Italy, Spain, other EU countries (Austria, Belgium, Bulgaria, Cyprus, Czech Republic, Denmark, Estonia, Finland, Greece, Hungary, Ireland, Latvia, Lithuania, Netherlands, Poland, Portugal, Romania, Slovakia, Slovenia, Sweden), UK, Brazil, Japan and China (grey areas represent the national lockdown periods in 2020).

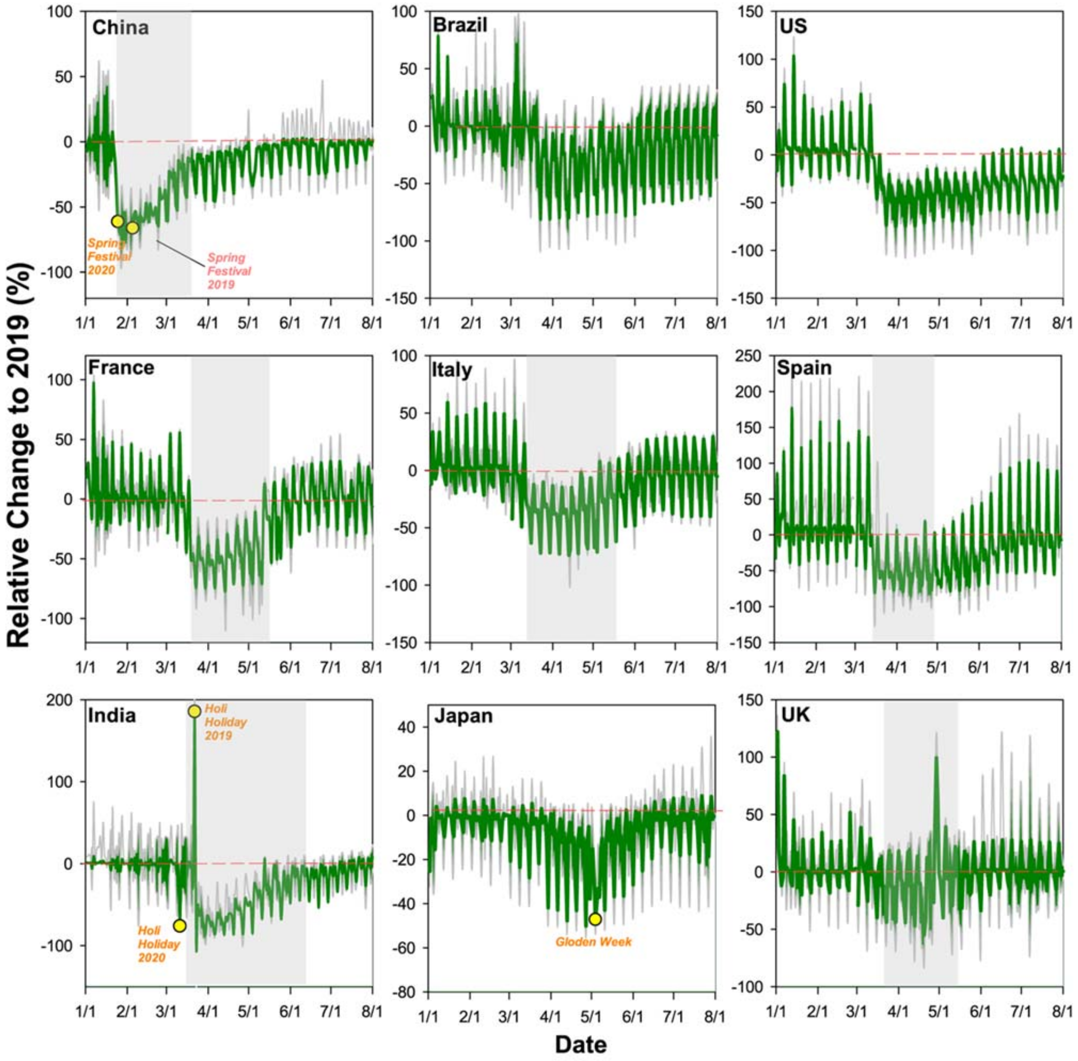

Supplementary Figure 2 | Daily CO_2 emission changes in the ground (road) transport sector. Daily emission changes in the ground (road) transport sector in the first half year of 2020 (green lines; grey lines for uncertainties; grey areas for the national lockdown periods in 2020).

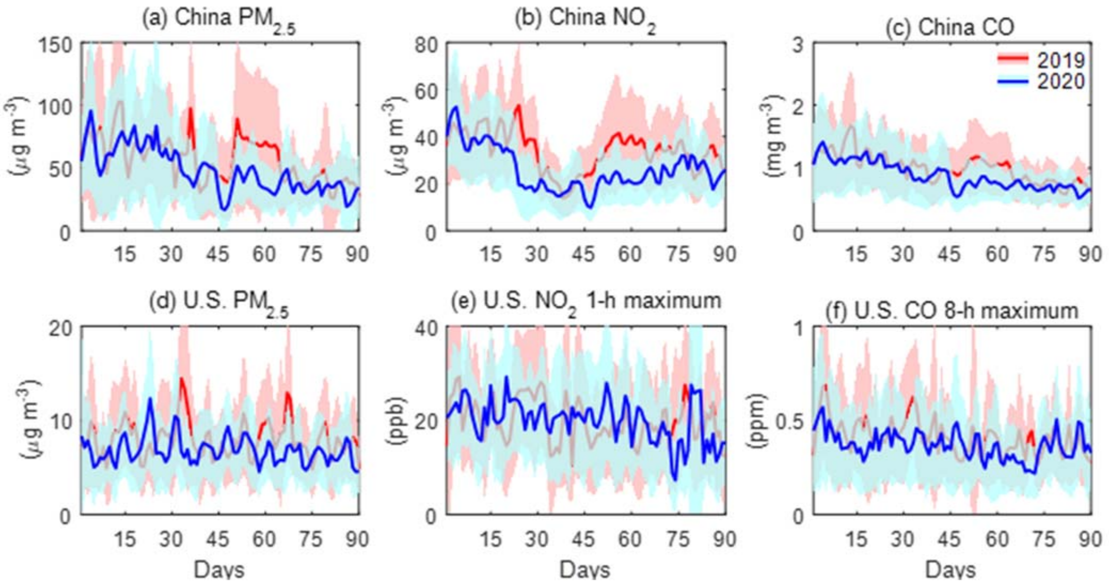

Supplementary Figure 3 | Daily variations of surface $PM_{2.5}$, NO_2 and CO concentrations. Daily variations of surface (a, d) $PM_{2.5}$, (b, d) NO_2, (c, f) CO concentrations from (a-c) China and (d-f) U.S. during the first quarters of 2019 and 2020. The bold lines are the mean values from all quality-controlled sites, with shadings indicating one standard deviation. The data on February 29th 2020 are removed from the plot.

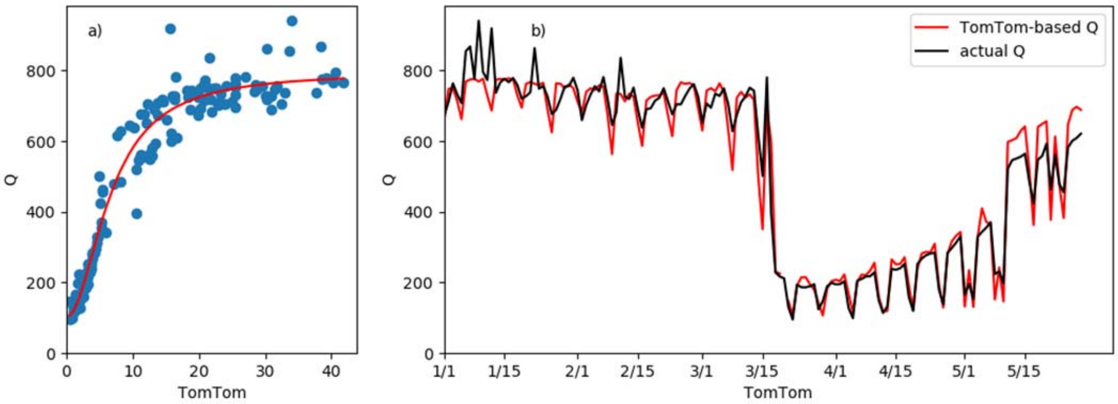

Supplementary Figure 4 | The relationship between TomTom congestion level with the daily mean car counts. The relationship between TomTom congestion index and the actual vehicle counts (Q in number of vehicles per hour each day) for Paris. a) the regression between TomTom congestion level (x-axis) and Q (y-axis); b) Q reconstructed based on TomTom congestion indexes (red) and the actual Q.

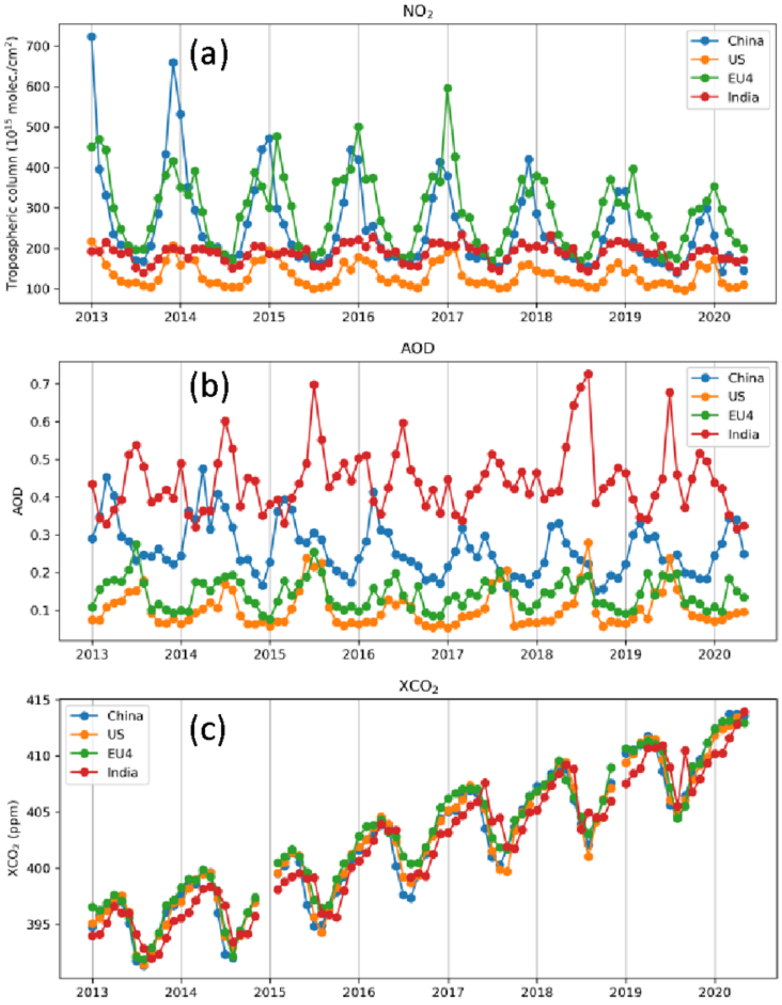

Supplementary Figure 5 | Monthly series of NO_2, aerosol optical depth (AOD) and column CO_2 mixing ratio (XCO_2). Monthly series of a) NO_2 from OMI, b) aerosol optical depth (AOD) from MODIS and c) column CO_2 mixing ratio (XCO_2) from GOSAT over China, US, selected EU countries (UK, Germany, Italy and France), and India.

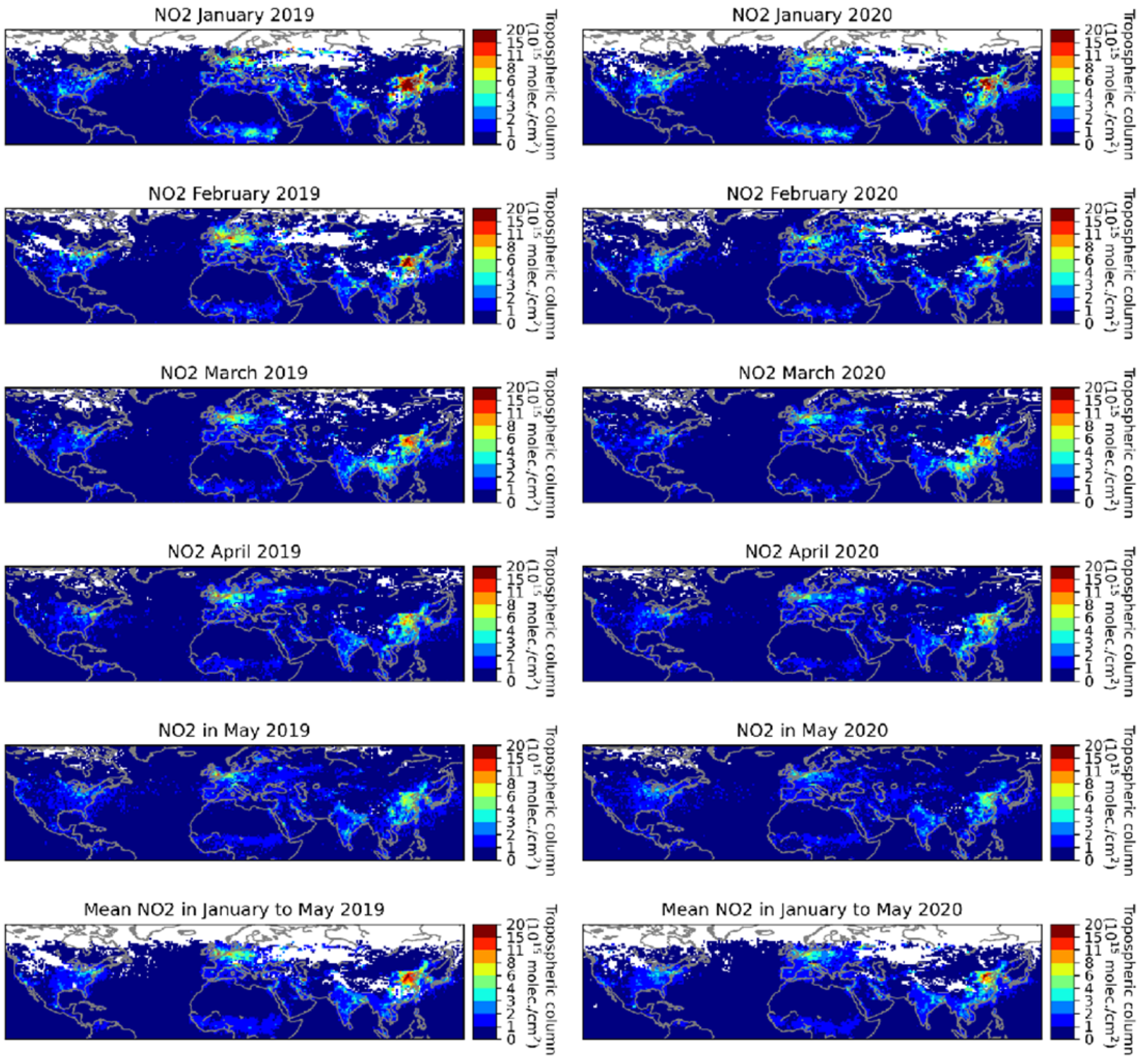

Supplementary Figure 6 | Tropospheric column NO_2 observation. Tropospheric column NO_2 observation in January - May of 2020. Source maps from GSHHG (Global Self-consistent, Hierarchical, High-resolution Geography Database)[1].

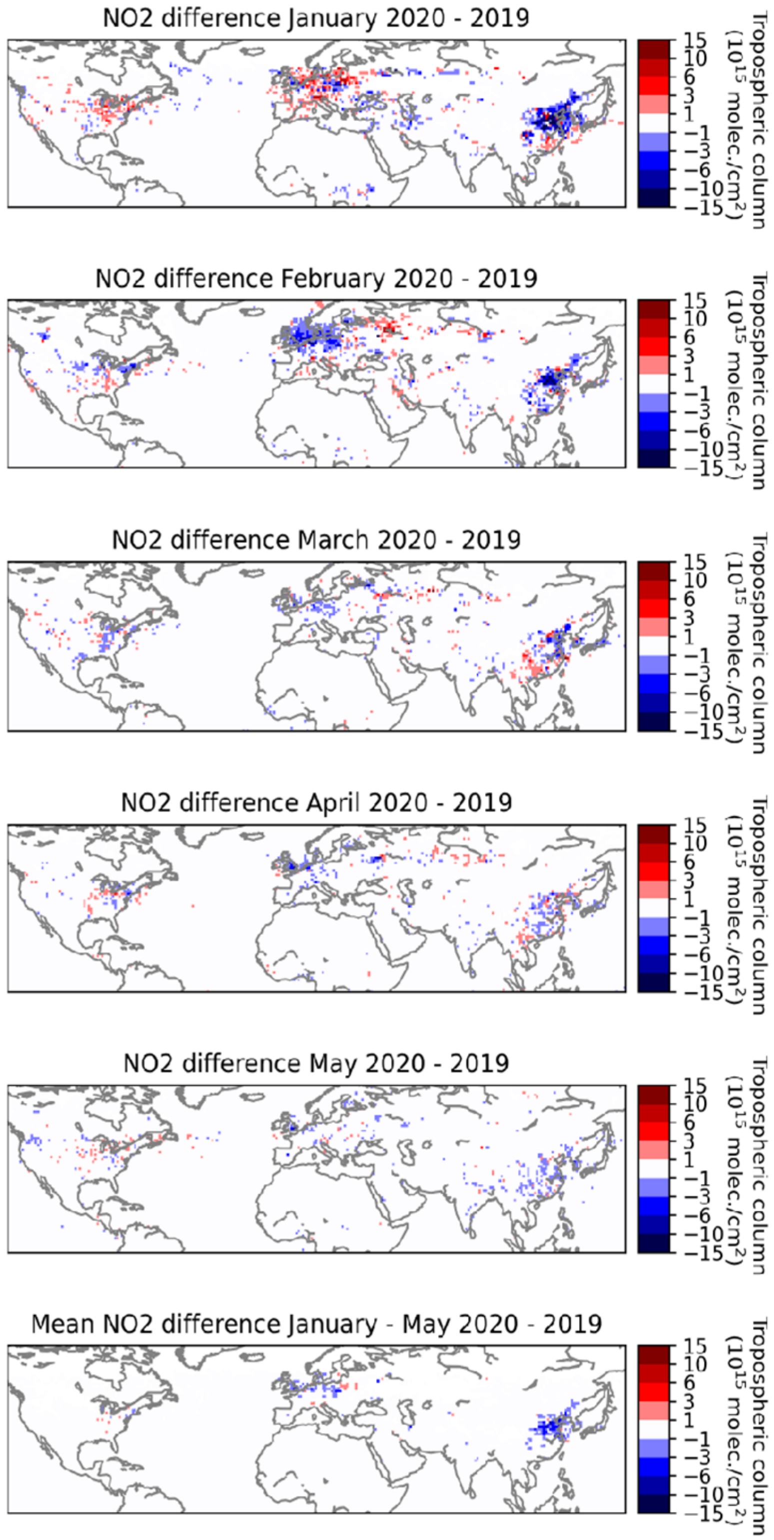

Supplementary Figure 7 | Difference of tropospheric column NO_2 observation. Difference of tropospheric column NO_2 observation in January - May of 2020. Source maps from GSHHG (Global Self-consistent, Hierarchical, High-resolution Geography Database) [1].

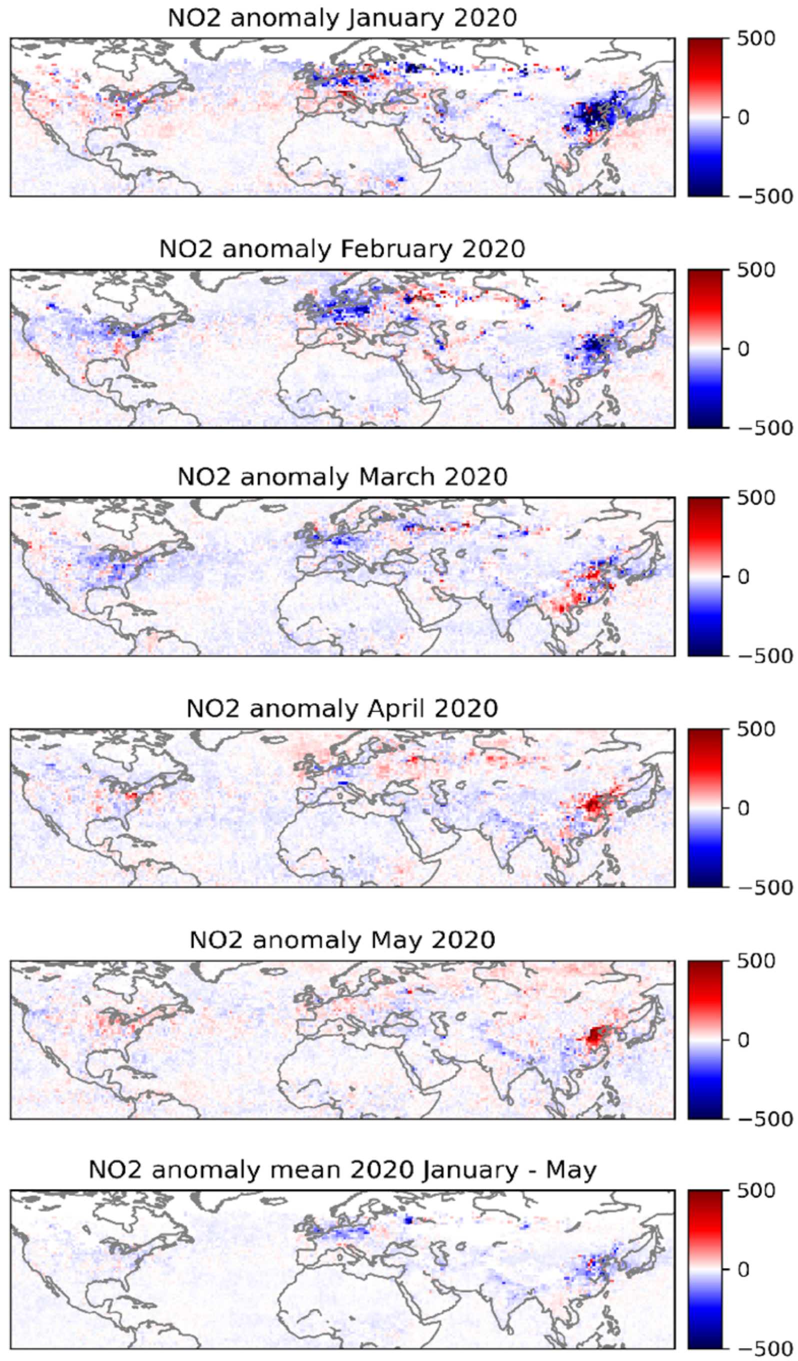

Supplementary Figure 8 | Anomaly of monthly NO_2. Anomaly of NO_2 from OMI in the January - May of 2020

The anomaly maps conducted by apply the same algorithm on every grid point. The anomaly defined as the deseasonalized value. For NO_2 (Supplementary Fig. 8), the anomaly along the eastern coast of China was negative in January and February 2020, then partially become positive. About half of the anomalies over U.S. and Europe were positive in January 2020, then most areas over U.S. and Europe became negative, which also matches the COVID-19 epidemic delays compared to China. Source maps from GSHHG (Global Self-consistent, Hierarchical, High-resolution Geography Database) [1].

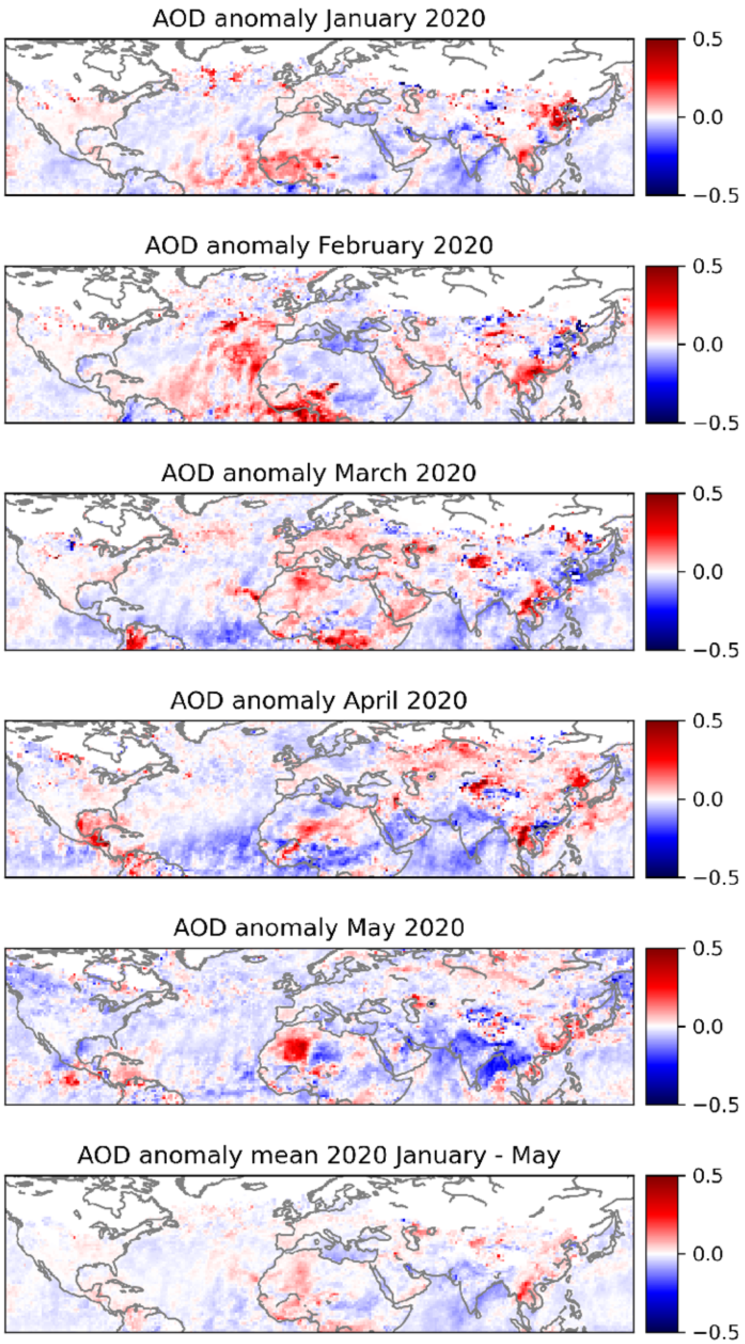

Supplementary Figure 9 | Anomaly of monthly AOD. Anomaly of AOD from MODIS in the January - May of 2020

The anomaly maps conducted by apply the same algorithm on every grid point. The anomaly defined as the deseasonalized value. For AOD (Supplementary Fig. 9), the negative anomaly area along the eastern coast of China expanded from January to March 2020. For US and Europe, AOD anomalies on land did not change too much. The shutdown of COVID-19 may not affect AOD over them since their AOD was always Low. Source maps from GSHHG (Global Self-consistent, Hierarchical, High-resolution Geography Database) [1]..

Supplementary Tables

Supplementary Table 1 | Sectoral changes of CO_2 emission (2020 compared to the same periods in 2019).

Unit: $MtCO_2$	Power	Ground Transport	Industry (with Process)	Residential	Domestic Aviation	Sum	Decline (%)
**China**	-31.3	-96.8	-40.0	-8.0	-11.1	-187.2	-3.7%
**India**	-83.6	-33.6	-92.6	7.0	-2.3	-205.2	-15.4%
**U.S.**	-66.3	-195.8	-36.5	-14.7	-24.9	-338.3	-13.3%
**EU27 & UK**	-98.5	-43.6	-43.5	-17.0	-3.1	-205.7	-12.7%
**Russia**	-20.6	-8.6	-3.3	-6.8	-1.2	-40.5	-5.3%
**Japan**	-16.0	-7.9	-16.2	-2.1	-1.0	-43.1	-7.5%
**Brazil**	1.3	-17.4	-8.3	0.0	-1.5	-25.9	-12.0%
**ROW**	-26.3	-209.6	-23.1	-0.9	-9.8	-269.6	-5.5%
**International Aviation**						-146.0	-48.5%
**International Shipping**						-89.1	-25.0%
**Sum**	-341.4	-613.3	-263.5	-42.5	-54.8	-1550.5	-8.8%
**Decline (%)**	-5.0%	-18.6%	-5.5%	-2.2%	-35.1%	-8.8%	

Supplementary Table 2 | Monthly changes of CO2 emissions in the power sector (2020 compared to the same periods in 2019).

	China	India	U.S.	EU27 & UK	Russia	Japan	Brazil	ROW	World
Jan	-3.6%	-0.3%	-9.7%	-18.8%	-1.8%	-6.2%	101.6%	0.0%	-4.2%
Feb	-14.4%	9.0%	-3.0%	-20.3%	-2.5%	-2.4%	-9.0%	3.8%	-5.1%
Mar	-8.0%	-12.8%	-9.0%	-10.8%	-6.5%	-3.6%	-16.7%	-2.7%	-7.1%
Apr	1.1%	-29.9%	-8.4%	-30.8%	-8.9%	-9.1%	-21.0%	-10.1%	-9.7%
May	11.5%	-20.7%	-13.9%	-26.3%	-6.6%	-12.8%	-11.5%	-0.8%	-3.3%
Jun	5.5%	-17.8%	-2.0%	-9.2%	-4.9%	-2.8%	5.0%	0.4%	-1.1%
Jan-Feb	-8.3%	4.1%	-6.6%	-19.5%	-2.1%	-4.4%	36.7%	1.7%	-4.6%
Jan-Mar	-8.2%	-1.9%	-7.4%	-17.0%	-3.6%	-4.2%	17.9%	0.3%	-5.4%
Jan-Apr	-6.0%	-9.2%	-7.6%	-19.9%	-4.8%	-5.2%	8.5%	-2.1%	-6.4%
Jan-May	-2.8%	-11.7%	-8.9%	-21.0%	-5.1%	-6.4%	4.7%	-1.8%	-5.8%
Jan-Jun	-1.4%	-12.7%	-7.6%	-19.3%	-5.1%	-5.9%	4.7%	-1.5%	-5.0%

Supplementary Table 3 | Monthly changes of CO_2 emissions in the industry sector (2020 compared to the same periods in 2019).

	China	India	U.S.	EU27 & UK	Russia	Japan	Brazil	ROW	World
Jan	-6.4%	1.8%	-0.7%	-1.2%	3.9%	-2.4%	1.5%	0.0%	-2.4%
Feb	-16.8%	3.8%	0.0%	-1.6%	4.9%	-5.6%	-0.4%	4.6%	-4.2%
Mar	-8.0%	-22.4%	-5.4%	-12.8%	2.6%	-5.3%	-4.2%	-2.2%	-7.3%
Apr	3.4%	-67.1%	-20.5%	-29.6%	-9.9%	-15.1%	-31.6%	-9.7%	-11.2%
May	4.4%	-39.3%	-16.6%	-21.6%	-7.2%	-26.3%	-23.7%	-1.1%	-5.4%
Jun	3.8%	-10.7%	-11.1%	-18.0%	-6.4%	-17.7%	-10.0%	0.0%	-2.0%
Jan-Feb	-10.9%	2.8%	-0.4%	-1.4%	4.4%	-4.0%	0.6%	2.1%	-3.2%
Jan-Mar	-9.7%	-6.1%	-2.1%	-5.2%	3.7%	-4.5%	-1.1%	0.5%	-4.7%
Jan-Apr	-5.9%	-20.5%	-6.7%	-11.3%	0.1%	-7.1%	-9.1%	-2.3%	-6.5%
Jan-May	-3.4%	-24.3%	-8.6%	-13.4%	-1.4%	-10.8%	-12.3%	-2.0%	-6.3%
Jan-Jun	-2.1%	-22.1%	-9.1%	-14.1%	-2.3%	-12.0%	-11.9%	-1.7%	-5.5%

Supplementary Table 4 | Monthly changes of CO_2 emissions in the ground transportation sector (2020 compared to the same periods in 2019).

	China	India	U.S.	EU27 & UK	Russia	Japan	Brazil	ROW	World
Jan	-18.6%	0.2%	7.7%	5.4%	6.1%	-3.0%	1.8%	3.2%	1.3%
Feb	-53.8%	1.9%	9.3%	7.1%	6.0%	0.9%	-0.2%	0.6%	-3.5%
Mar	-25.0%	-25.7%	-23.5%	-16.7%	-3.2%	-7.1%	-15.2%	-22.2%	-21.0%
Apr	-16.1%	-65.6%	-49.1%	-31.9%	-26.1%	-17.4%	-37.7%	-41.9%	-38.6%
May	-10.8%	-34.4%	-45.7%	-20.7%	-20.4%	-18.4%	-34.2%	-37.9%	-32.6%
Jun	-5.9%	-16.7%	-30.2%	-1.2%	-4.9%	-4.0%	-19.0%	-14.1%	-15.2%
Jul	-4.2%	-7.2%	-30.9%	-0.3%	-3.5%	-4.0%	-15.6%	-9.3%	-13.0%
Jan-Feb	-35.4%	1.0%	8.5%	6.2%	6.1%	-1.1%	0.8%	1.9%	-1.0%
Jan-Mar	-31.8%	-8.1%	-2.7%	-1.8%	2.8%	-3.2%	-4.8%	-6.5%	-8.0%
Jan-Apr	-27.9%	-22.3%	-14.7%	-9.4%	-4.6%	-6.7%	-13.2%	-15.4%	-15.7%
Jan-May	-24.4%	-24.8%	-21.2%	-11.7%	-7.9%	-9.1%	-17.6%	-20.1%	-19.2%
Jan-Jun	-21.4%	-23.5%	-22.7%	-10.0%	-7.4%	-8.3%	-17.8%	-19.2%	-18.6%
Jan-Jul	-18.9%	-21.1%	-24.0%	-8.5%	-6.8%	-7.7%	-17.5%	-17.8%	-17.8%

Supplementary Table 5 | Monthly changes of CO_2 emissions in the aviation Sector (2020 compared to the same periods in 2019).

Month	Domestic									International	World
	China	India	U.S.	EU27 & UK	Russia	Japan	Brazil	ROW	All		
Jan	2.4%	4.4%	8.5%	0.0%	14.4%	7.2%	6.4%	7.9%	6.8%	3.7%	4.8%
Feb	-71.2%	11.6%	12.1%	2.7%	13.1%	10.3%	9.5%	9.2%	-5.4%	-3.0%	-3.8%
Mar	-56.4%	-18.8%	-13.2%	-44.6%	6.1%	-4.0%	-18.3%	-20.3%	-22.7%	-39.5%	-33.6%
Apr	-51.7%	-98.6%	-65.4%	-90.9%	-65.6%	-47.3%	-87.7%	-80.5%	-67.6%	-83.5%	-78.1%
May	-34.9%	-93.7%	-64.4%	-87.9%	-64.1%	-75.3%	-83.2%	-77.9%	-63.8%	-78.8%	-73.7%
Jun	-24.6%	-70.8%	-53.4%	-76.5%	-36.5%	-61.0%	-76.0%	-64.2%	-51.8%	-78.5%	-69.6%
Jul	-14.7%	-70.3%	-41.9%	-48.8%	-9.8%	-36.9%	-69.7%	-54.1%	-39.6%	-72.0%	-61.3%
Jan-Feb	-33.7%	7.8%	10.2%	1.3%	13.8%	8.7%	7.8%	8.6%	0.9%	0.5%	0.7%
Jan-Mar	-41.2%	-1.1%	1.6%	-15.2%	11.1%	4.3%	-0.5%	-1.5%	-7.4%	-13.5%	-11.4%
Jan-Apr	-43.8%	-24.0%	-15.9%	-35.6%	-9.1%	-8.5%	-20.4%	-21.6%	-22.8%	-31.8%	-28.7%
Jan-May	-42.0%	-38.4%	-26.4%	-47.2%	-21.5%	-22.5%	-31.7%	-33.4%	-31.5%	-41.8%	-38.3%
Jan-Jun	-39.1%	-43.9%	-31.3%	-52.7%	-24.5%	-29.0%	-38.4%	-38.8%	-35.1%	-48.5%	-43.9%
Jan-Jul	-35.3%	-47.8%	-33.0%	-52.0%	-21.9%	-30.2%	-43.2%	-41.2%	-35.8%	-52.4%	-46.7%

Supplementary Table 6 | The observation of air quality and dry column CO_2 (XCO_2) (2020 compared to the same periods in 2019).

		China	U.S.	EU4	India
**OMI NO_2**	**Jan**	-32.26% ± 11.93%	22.98% ± 15.94%	15.78% ± 15.39%	-8.96% ± 13.55%
	**Feb**	-34.22% ± 12.00%	-23.08% ± 12.45%	-25.12% ± 12.52%	-13.79% ± 13.16%
	**Mar**	-4.53% ± 13.73%	-14.32% ± 13.16%	-15.56% ± 13.15%	-13.37% ± 13.17%
	**Apr**	-3.59% ± 13.97%	-2.16% ± 13.89%	-23.40% ± 12.52%	-11.10% ± 13.40%
	**May**	-12.63% ± 13.40%	-1.25% ± 13.98%	-12.49% ± 13.37%	-8.12% ± 13.57%
	**Jan-May**	-20.18% ± 12.99%	-3.70% ± 13.93%	-12.69% ± 13.20%	-11.09% ± 13.43%
**MODIS AOD**	**Jan**	10.17% ± 48.48%	10.64% ± 95.09%	19.81% ± 77.91%	-5.46% ± 36.66%
	**Feb**	-7.88% ± 41.27%	-3.98% ± 82.63%	-1.95% ± 70.87%	7.29% ± 40.64%
	**Mar**	3.55% ± 41.33%	-15.65% ± 66.76%	29.42% ± 62.13%	1.84% ± 40.56%
	**Apr**	17.35% ± 45.72%	20.16% ± 86.26%	-23.83% ± 46.27%	-7.65% ± 39.46%
	**May**	-15.88% ± 40.58%	-35.61% ± 51.83%	-3.74% ± 58.60%	-19.66% ± 36.57%
	**Jan-May**	0.97% ± 43.26%	-10.54% ± 71.89%	0.83% ± 59.73%	-4.91% ± 38.20%
**GOSAT XCO_2**	**Jan**	0.53% ± 0.52%	0.60% ± 0.52%	0.42% ± 0.52%	0.65% ± 0.52%
	**Feb**	0.45% ± 0.52%	0.53% ± 0.52%	0.65% ± 0.52%	0.44% ± 0.52%
	**Mar**	0.67% ± 0.52%	0.37% ± 0.52%	0.51% ± 0.51%	0.66% ± 0.53%
	**Apr**	0.48% ± 0.52%	0.46% ± 0.51%	0.39% ± 0.52%	0.51% ± 0.52%
	**May**	0.66% ± 0.52%	0.59% ± 0.52%	0.45% ± 0.51%	0.78% ± 0.52%
	**Jan-May**	0.56% ± 0.52%	0.51% ± 0.51%	0.48% ± 0.51%	0.61% ± 0.52%
**TROPOMI CO**	**Jan**	2.67%±5.20%	4.94%±3.02%	2.37%±1.89%	-0.41%±3.37%
	**Feb**	0.47%±7.11%	2.97%±3.91%	1.08%±2.33%	3.23%±5.20%
	**Mar**	3.98%±6.77%	-1.84%±3.39%	-1.14%±3.10%	2.12%±3.45%
	**Jan-Mar**	2.38%±4.84%	1.85%±1.94%	0.72%±1.40%	1.66%±2.38%
**Site NO_2**	**Jan**	-18.05%±23.90%	-3.80%±12.80%		
	**Feb**	-30.33%±21.78%	14.98%±68.82%		
	**Mar**	-23.03%±17.29%	-8.98%±44.17%		
	**Jan-Mar**	-23.00%±14.97%	0.34%±79.05%		
**Site $PM_{2.5}$**	**Jan**	-2.67%±41.56%	-8.77%±49.43%		
	**Feb**	-26.71%±26.94%	-14.78%±59.12%		
	**Mar**	-21.80%±17.51%	-20.55%±39.30%		
	**Jan-Mar**	-15.39%±19.06%	-14.68%±40.49%		
**Site CO**	**Jan**	-5.89%±22.22%	-12.35%±23.12%		
	**Feb**	-19.60%±20.49%	-6.19%±45.39%		
	**Mar**	-14.24%±19.77%	4.94%±74.21%		

	**Jan-Mar**	-12.51%±15.41%	-5.11%±26.53%
**Inventory NO_2**	**Jan**		-0.99% (-1.36~-0.86%)
	**Feb**		2.43% (2.11~3.33%)
	**Mar**	-15.49% (-21.20~-13.46%)	-7.72% (-10.58~-6.72%)
	**Jan-Mar**	-17.47% (-23.94~-15.20%)	-2.57% (-3.52~-2.24%)

Supplementary Table 7 | Percentage uncertainty for daily emission 2020

Items	Uncertainty Range
Power	±14.0%
Ground transport	±9.3%
Industry	±36.0%
Residential	±40.0%
Aviation	±10.2%
International shipping	±13.0%
Projection of emissions growth rate in 2019	±0.8%
EDGAR emissions in 2018	±5.0%
**Overall**	**±7.2%**

Supplementary Note 1 Comparison of Liu et al. and Le Quéré et al.

As requested by reviewer and editor, we compared our study with the recently published work[2] by Le Quéré et al. that addressed a similar topic although with a different approach when looking in details:

In short:

Le Quéré et al. relied on confinement index intensity to attribute changes of CO_2 emissions due to COVID-19 pandemic. Le Quéré et al. assumed that emissions reductions scaled linearly according to activity data for selected periods / countries and established relationships between % change activity data and confinement indexes severity. These relationships were then applied with daily confinement indexes to infer emission changes per sector / country.

In this approach, only COVID (confinement) effects on emissions were modeled, not effects of other factors which also drove actual daily emissions in 2020: changes of weather (cold / mild winter) or in energy mix for power production, due to low gas price at the same time as the confinement. When confinement levels returned to zero, real emissions can be reduced or increased and by construction the approach of Le Quéré et al. cannot track those changes.

In our study, we used daily activity data to quantify emissions for all sectors, which allows continuing to track daily emission dynamics after the end of the lockdowns. At face value, our approach gives an assessment of actual emissions changes from all factors, including dominant effects of COVID during the lockdown but also effect of warm winter weather, rebounds of industry emissions after confinements, continued depletion of transport emissions (especially aviation), and changes of energy mix (power sector emissions). Our methodology also captures emission reductions during holidays when they happen, which can be on different days of the year across different years.

In more details:

We document below some key methodological differences.

Traffic emissions: Le Quéré et al. traffic emissions were scaled linearly with 7-days traffic TomTom congestion indices, supplemented by data from data from Apple (58 countries), the US MS2 corporation (20 states) and the UK government. Their approach was to take % changes of those indices in the week of April 4 at discrete confinement levels (as defined by policies in place) across the available datasets, and then apply this relationship (% changes of traffic indices vs discrete confinement level) according to daily confinement index in each country. The discrete nature of confinement index values explains why there are ‘steps’ in daily changes reconstructed from Le Quéré et al. when the index moves from one discrete value to another.

Instead, we used directly TomTom indices as a continuous variable because we accessed daily data in several cities within each country. We showed this index was a nonlinear function of the actual car flux (thus emissions) and calibrated and applied this nonlinear function to infer daily emissions from each country.

Residential emissions: Le Quéré et al. estimates were based on confinement indexes and electricity consumption for the city of London (assuming implicitly that electricity consumption may scale with fuel use in buildings). In our study, residential emissions are assumed to depend on temperature in cold countries and were estimated based on 2019 fuel consumption data with established temperature functions of temperature in 2019 and 2020. We adopted this approach after verifying that there has been no effect of confinement severity on residential emissions by analyzing actual daily natural gas residential consumption data in EU countries, where such data were available.

Power sector: Le Quéré et al. used daily electricity demand data and did not indicate energy mix changes coincident to the COVID period and used power production dataset for US, India and European Total (see table below) corrected for temperature. We included daily energy mix changes by using thermal production data to calculate power sector emissions and consider the changes of fuel mix in thermal production in the uncertainties, with data for 31 countries. Our emission estimates are provided as actual values, not corrected for temperature but we also provide attribution to temperature vs. COVID in the manuscript.

Aviation emissions Le Quéré et al. used weekly OAG global flight numbers. We used individual flight data (thus daily) split into countries between domestic and international emissions

Industry fuel use: Le Quéré et al. used total coal consumption and we used actual production data for China. Le Quéré et al. used confinement indexes for other countries. We used production indexes for the other countries and confinement in ROW.

Importantly, Le Quéré's paper indicated the urgent need and research gap of the real time CO_2 study, which has exactly been addressed by our research:

"Despite the critical importance of CO_2 emissions for understanding global climate change, **systems are not in place to monitor global emissions in real time**." (page 1 paragraph 3 in Le Quéré paper[2])

Supplementary Table 8 | Comparison of Liu et al. paper and Le Quéré et al. paper

		Liu et al. paper	Le Quéré et al. paper
**Results**	Missions for each day	Included for 2019 and January-June 2020 based on continuous daily activity data with some sectors updated to July	Daily emission decline caused by COVID only based on relationship between confinement levels and activity data for the period January-April 2020 (duration of confinement period)
	Weekly difference	Considered	N/A
	Seasonality	Considered whole year 2019 and 2020 seasonality	Considered by HDD for power sector during January-April 2020
	Holidays	Considered	N/A
	Changes in the energy mix or e.g. the fossil/renewable ratio	Considered	N/A
	Temperature effects on power and residential emissions	Considered for both sectors	Considered for power sector
**Method**	Total	Estimated daily emissions based on actual daily activity data in each country	Confinement index based: establish % activity change at each confinement level across the available datasets, and then apply those % changes according to confinement levels in place on each day in each country
	Sector-Power	Hourly to daily thermal production for 31 countries including variable fuel mix; Temperature effects separated for each country	Relationship between confinement levels and daily temperature corrected electricity generation data in U.S., European countries (as a total), and India
	Sector-Industry	Emissions based on industrial production or industry output index data in each country	Confinement index based on daily coal consumption data for the six largest coal companies in China
	Sector-Surface Transport	TomTom congestion Index-Emission' model calibrated against car flux data (in	Confinement index based: establish fixed % traffic proxies (TomTom, Apple, US MS2, UK)

		Paris). This emission model evaluated for few other cities	and changes at each confinement level across the available datasets, and then apply those % changes according to confinement levels in place on each day in each country
	Sector-Commercial and Residential	Population-weighted heating degree days in 206 countries assuming no direct effect of COVID on sector’s fuel consumption<br><br>This assumption was partly verified by daily natural gas consumption by commercial and residential buildings in France, Italy, Belgium and Spain	Public: estimated by the changes in transport sector and electricity sector<br><br>Residential: emission changes inferred from residential electricity use monitored with UK smart meters
	Sector-Aviation	Daily individual flight distance with each aircraft type by Flight Radar24	weekly flight numbers by OAG
	Sector-Shipping	Decline ratio by reports	Projections of the World Trade Organization of -20% regardless of the confinement level
**Data**	Sector-Power	31 countries	(US, India and European Total)
	Sector-Industry	62 countries	China (six power companies) and US
	Sector-Surface Transport	57 countries and 416 cities	63 countries and 413 cities
	Sector-Commercial and Residential	206 countries	UK
	Sector-Aviation	All countries	14 countries
	Sector-Shipping	International Shipping (only global total)	International Shipping (only global total)

Supplementary References:

1. Wessel, Pål; Smith, Walter H. F. A global, self-consistent, hierarchical, high-resolution shoreline database. Journal of Geophysical Research: Solid Earth. 101 (B4): 8741–8743(1996). doi:10.1029/96JB00104

2. Le Quéré, C., Jackson, R.B., Jones, M.W. et al. Temporary reduction in daily global CO2 emissions during the COVID-19 forced confinement. Nat. Clim. Chang. 10, 647–653 (2020). https://doi.org/10.1038/s41558-020-0797-x
